# Supplementary material for: Multiple sclerosis: Exploring the limits and implications of genetic and environmental susceptibility
Source: PLoS One. 2023 Jun 28;18(6):e0285599. doi: 10.1371/journal.pone.0285599 (PMC10306391; doi:10.1371/journal.pone.0285599)
Supplement: S1 File — (PDF) [file pone.0285599.s001.pdf]

Supplemental Material

1. *Adjusting the MZ-twin Concordance for the Shared (E_{twn}) Environment*

1a. Defining the Adjusted MZ-twin Concordance {$P(MS \mid IG_{MS})$ …………………………… p. 2

1b. Estimating the Magnitude of the Necessary Adjustment ……………………………… p. 3

1c. Adjustment for the Susceptible Women and Men Considered Together .... p. 6

1d. Adjustment for the Susceptible Women and Men Considered Separately .... p. 7

2. *Enrichment of Genotypes*

2a. Enrichment from the subset (G) to the subset (MS) .... p. 8

2b. Enrichment from the subset (G, MS) to the subset (G, MS, MZ_{MS}) .... p. 9

2c. Current Enrichment of Women Compared to Men .... p. 9

3. *Cross-sectional Model*

3a. Model Development .... p. 11

3b. Assertion C Solutions ……………………………………………………………………….p. 16

3c. Quadratic Solutions for Women and Men ………………………………………………….p. 16

4. *Longitudinal Model*

4a. Model Development .... p. 18

4b. Environmental Exposure Levels during Different Time Periods .... p. 19

4c. Relationships for the Susceptible Population as a Whole .... p. 21

4d. Relationship of Failure to True Survival .... p. 22

4e. Relationship of the (F:M) Sex Ratio to Exposure .... p. 23

4f. Response Curves to Increasing Exposure .... p. 24

5. *Non-proportional Hazard Models*

5a. General Considerations .... p. 24

6. *Proportional Hazard Models*

6a. General Considerations .... p. 25

6b. Defining an "Apparent" Proportionality Factor .... p. 26

6c. Implications that the (R) Value has for the Values of (λ), ($\boldsymbol{c}$) and ($\boldsymbol{d}$) .... p. 27

6d. Strictly Proportional Hazard: ($\lambda = 0$) .... p. 29

6e. Intermediate Proportional Hazard: ($\lambda < 0$) .... p. 30

6f. Intermediate Proportional Hazard: ($\lambda > 0$) .... p. 30

6g. Considerations of Exposure "Intensity" .... p. 31

6h. Exposure Variability (i.e., for R_i & λ_i) among "i-type" Individuals .... p. 38

7. *Summary Equations for the Longitudinal Model*

7a. Derivations .... p. 40

7b. Limits on the Value of the Parameters: $P(MS \mid E)$, ($\boldsymbol{c}$) and ($\boldsymbol{d}$) .... p. 41

8. *Figures S1–S3*

8a. General (Common) Considerations for the Figures ……………………………..................... p. 42

8b. Figures S1–S3 .... pp. 44–46

1. *Adjusting the MZ-twin Concordance for the Shared* (E_{twn}) *Environment*

1a. *Defining the Adjusted MZ-twin Concordance* $\{P(MS \mid IG_{MS})\}$

Because, by definition (*see Methods #1A & B; Main Text*):

$$P(MS) = P(MS, E) = P(MS, E, G)$$

therefore: $P(MS \mid MZ_{MS}) = P(MS, E, G \mid MZ_{MS}) = \sum_{i=1}^{m} P\big(MS, E, G_i \mid MZ_{MS}\big)$

where: $\forall(i)\colon\ (i = 1,2,\ldots,m)$:

$$P(MS, E, G_i \mid MZ_{MS} = P(MS \mid E, G_i, MZ_{MS}) * P(E \mid G_i, MZ_{MS}) * P(G_i \mid MZ_{MS})$$

In this way, the probability of the proband being a member of the (MS, E, G_i) subset, given the fact that their co-twin is a member of the (MS, MZ) subset – i.e., $\{P(MS, E, G_i \mid MZ_{MS})\}$ – can be re-expressed as the product of three component probabilities – the probability of MS developing in an *MZ*-proband (G_i) who experiences a "*sufficient*" environment, the probability that this *MZ*-proband experiences an environment "*sufficient*" to cause MS, and the probability of this *MZ*-proband being a member of the (G_i) subset – where each probability is conditioned on the proband having an *MZ* co-twin, who is a member of the (MS, MZ) subset within (Z).

For susceptible probands who are members of (G), but who are not otherwise identified, the analogous probabilities can be written:

$$P(MS \mid G) = P(MS, E, G \mid G) = \sum_{i=1}^{m} P\big(MS, E, G_i \mid G\big)$$

where: $\forall(i)\colon\ (i = 1,2,\ldots,m)$:

$$P(MS, E, G_i \mid G) = P(MS \mid E, G_i) * P(E \mid G_i) * P(G_i \mid G)$$

Therefore, to estimate the desired "adjusted" probability, $P(MS \mid IG_{MS})$, we need to remove the impact of the shared environment of *MZ*-twins, while leaving the genetic impact of being *MZ*-twins unchanged. Thus, we define:

$$P(MS, E, G_i \mid IG_{MS}) = P(MS \mid E, G_i, IG_{MS}) * P(E \mid G_i, IG_{MS}) * P(G_i \mid IG_{MS})$$

where: $P(E \mid G_i, IG_{MS}) = P(E \mid G_i)$

and: $P(G_i \mid IG_{MS}) = P(G_i \mid MZ_{MS})$

Moreover, we note that the conditioning events (E, G_i) and (E, G_i, MZ_{MS}) both represent the same underlying event for the proband – i.e., the event that the *i*th susceptible individual (the *MZ*-proband) experiences an environment "*sufficient*" to cause MS in them. In this circumstance, therefore:

$$P(MS \mid E, G_i, MZ_{MS}) = P(MS \mid E, G_i, IG_{MS}) = P(MS \mid E, G_i)$$

Incorporating these equivalences, into the *above* definition of (IG_{MS}), yields:

$$P(MS, G_i \mid IG_{MS}) = P(MS, E, G_i \mid IG_{MS}) = P(MS \mid E, G_i) * P(E \mid G_i) * P(G_i \mid MZ_{MS})$$

or: $$P(MS, G_i \mid IG_{MS}) = P(MS \mid G_i) * P(G_i \mid MZ_{MS}) = (x_i) * P(G_i \mid MZ_{MS})$$

In this manner, to summarize the *above* arguments, the term (IG_{MS}) is defined such that:

$\forall(i)$: $(i = 1,2, \dots, m)$:

$$\frac{P(MS, G_i \mid IG_{MS})}{P(G_i \mid IG_{MS})} = P(MS \mid G_i, IG_{MS}) = P(MS \mid G_i) = x_i$$

and: $$P(G_i \mid IG_{MS}) = P(G_i \mid MZ_{MS})$$

Therefore, the desired "adjusted" probability, $P(MS \mid IG_{MS})$, can be expressed such that:

$$P(MS \mid IG_{MS}) = \sum_{i=1}^{m} P(MS, G_i \mid IG_{MS}) = \sum_{i=1}^{m} P(G_i \mid MZ_{MS}) * (x_i)$$

Effectively, this adjustment equates to a thought-experiment, in which susceptible *MZ*-twins are separated at conception, and in which the proband twin experiences the (E_{twn}), (E_{sib}), and (E_{pop}) environments just as would any other member of the (G) subset, given the environmental conditions of the time (E_T) – *see Methods #1E (Main Text)* for a description of this partition. In addition, as discussed previously (*Methods #1B*), the available evidence suggests that the (E_{sib}) environment has no impact on the likelihood of a susceptible individual subsequently developing MS [10-16]. If this evidence is correct, it will also be the case that:

$$P(E \mid G_i, IG_{MS}) = P(E \mid G_i, S_{MS}) = P(E \mid G_i)$$

{NB: This definition represents the intended meaning of the "adjusted" proband-wise recurrence rate for MZ-twins – i.e., $P(MS \mid IG_{MS})$*. The next section is devoted to estimating an adjustment that matches this definition and that is derived from directly observed epidemiological data.}*

1b. Estimating the Magnitude of the Necessary Adjustment

Assertions: $$s_a = P(MS \mid DZ_{MS}, E_T)/P(MS \mid S_{MS}, E_T)$$

$$P(MS \mid IG_{MS}, E_T) = P(MS \mid MZ_{MS}, E_T)/s_a$$

Proof: Here the event of interest is the development of MS in a randomly selected proband *MZ*-twin, whose co-twin already has or will develop MS – i.e., the co-twin of this proband is a member of the (MZ_{MS}) subset. Consequently, in this conceptualization, the event (IG_{MS}), which is independent of whatever happens to the proband, is envisioned to be identical to the event (MZ_{MS}), defined *above*. Therefore, during any specific *Time Period*, (E_T):

$$P(IG_{MS} \mid E_T) = P(MZ_{MS} \mid E_T)$$

As discussed *above* and in the *Main Text* (*Methods #1D*), however, the probability of this event – i.e., $P(MS \mid IG_{MS})$ – may differ from $P(MS \mid MZ_{MS})$, due to the shared environmental experiences of *MZ*-twins.

Including the circumstances of both twins and siblings, the occurrences of: (MZ_E), (DZ_E), and (S_E) represent, respectively, the events that a co-*MZ*-twin, a co-*DZ*-twin, or a non-twin co-sibling, experience the exposure (E). The respective probabilities that the probands of these co-twins or co-siblings experience $\{E_i\}$, given that they are members of the (G) subset, are expressed as: $P(\{E_i\} \mid MZ_E)$; $P(\{E_i\} \mid G, DZ_E)$; and: $P(\{E_i\} \mid G, S_E)$. Moreover, because any two siblings and any set of *DZ*-twins have the same genetic relationship to each other, and because, by definition, they both disproportionately share the same (E_{sib}) environment, the only difference between them is that *DZ*-twins share the same (E_{twn}) environment whereas siblings don't.

These probabilities are then contrasted to the probability of the event $\{E_i\}$ for a randomly selected member of the (G) subset – i.e., $P(\{E_i\} \mid G)$. As *above*, we can partition each of these exposure events into three independent component events $(E_{twn}, E_{sib}, \ \& \ E_{pop})$, during any *Time Period*, such that:

$$\forall(i): \ (i = 1,2, \dots, m):$$

$$P(\{E_i\} \mid MZ_E) \ = P(\{E_{twn}\}_i \mid MZ_E) * P(\{E_{sib}\}_i \mid G, S_E) * P(\{E_{pop}\}_i \mid G)$$

$$P(\{E_i\} \mid G, DZ_E) = P(\{E_{twn}\}_i \mid G, DZ_E) * P(\{E_{sib}\}_i \mid G, S_E) * P(\{E_{pop}\}_i \mid G)$$

$$P(\{E_i\} \mid G, S_E) = P(\{E_{twn}\}_i \mid G) * P(\{E_{sib}\}_i \mid G, S_E) * P(\{E_{pop}\}_i \mid G)$$

and: $$P(\{E_i\} \mid G) = P(\{E_{twn}\}_i \mid G) * P(\{E_{sib}\}_i \mid G) * P(\{E_{pop}\}_i \mid G)$$

Probands both from an *MZ*-twinship and those from a *DZ*-twinship experience the same (E_{twn}) environment as their co-twin who either has or will develop MS. Therefore, we assume that:

$$P(\{E_{twn}\}_i \mid MZ_E) = P(\{E_{twn}\}_i \mid G, DZ_E)$$

so that: $$P(\{E_i\} \mid MZ_E) = P(\{E_i\} \mid G, DZ_E)$$

In this circumstance:

$$P(\{E_i\} \mid G, DZ_E)/P(\{E_i\} \mid G, S_E) = P(\{E_{twn}\}_i \mid G, DZ_E)/P(\{E_{twn}\}_i \mid G) = B_i \geq 1$$

so that: $$P(\{E_i\} \mid G, S_E) = P(\{E_i\} \mid G, DZ_E)/B_i$$

where $(B_i \geq 1)$ is a constant for each (i) during any specific *Time Period* (E_T).

The terms $P(MS, G_i, \{E_i\} \mid G, DZ_E)$ and $P(MS, G_i, \{E_i\} \mid G, S_E)$ represent, respectively, the probabilities of the events that the ith individual, randomly selected from (Z), is a member of either the (MS, DZ) or the (MS, S) subsets, given that they are a member of the (G) subset and also given that their co-twin or co-sibling is a member of either the (DZ, E) or the (S, E) subsets. However, by definition (*see Methods*

#1B; Main Text), if both events (G) and $\{E_i\}$ occur together, then the event (MS, G_i) will either occur or not occur, independently of the status of a co-twin or co-sibling.

Therefore, $\forall(i)$: $(i = 1,2, \ldots, m)$:

$$P(MS, G_i \,|\, \{E_i\}, MZ_E) = P(MS, G_i \,|\, \{E_i\}, G, DZ_E) = P(MS, G_i \,|\, \{E_i\}, G, S_E) = P(MS, G_i \,|\, \{E_i\}, G)$$

and, thus: $\frac{P(MS,G_i,\{E_i\} \,|\, G,DZ_E)}{P(MS,G_i,\{E_i\} \,|\, G,S_E)} = \frac{P(MS,G_i \,|\, \{E_i\},G,DZ_E)}{P(MS,G_i \,|\, \{E_i\},G,S_E)} * \frac{P(\{E_i\} \,|\, G,DZ_E)}{P(\{E_i\} \,|\, G,S_E)} = \frac{P(\{E_i\} \,|\, G,DZ_E)}{P(\{E_i\} \,|\, G,S_E)} = B_i \geq 1$

In this manner, we can use the adjustment factor (B_i), to remove, mathematically, the impact of the shared $\{E_{twn}\}$ environment for the ith individual, during any *Time Period*, such that:

$$P(MS, G_i, \{E_i\} \,|\, G, S_E) = P(MS, G_i, \{E_i\} \,|\, G, DZ_E)/B_i$$

where, because: $\sum_{i=1}^{m} P(MS, G_i, \{E_i\} \,|\, G, S_E) = P(MS, E \,|\, G, S_E)$

therefore: $P(MS, E \,|\, G, S_E) = \sum_{i=1}^{m} P(MS, G_i, \{E_i\} \,|\, G, DZ_E)/B_i$

In this case, we can assign variables (y) and (z) such that:

$$y = 1/B_i \quad \text{and:} \quad z = P(MS, G_i, \{E_i\} \,|\, G, DZ_E)$$

In which case, the covariance (σ_{yz}), using its standard definition [39], can be expressed such that:

$$\sigma_{yz} = E(yz) - E(y) * E(z)$$

where: $\sum_{i=1}^{m} P(MS, G_i, \{E_i\} \,|\, G, DZ_E)/B_i = m * E(yz)$

and where: $\sum_{i=1}^{m} P(MS, G_i, \{E_i\} \,|\, G, DZ_E) = m * E(z) = P(MS, E \,|\, G, DZ_E)$

Because (*from above*): $\forall(i) : B_i \geq 1$

Therefore: $m * E(yz) \leq m * E(z)$

In this circumstance, we can define an adjustment factor (s_a) such that:

$$E(yz) = E(z)/s_a \leq E(z)$$

where: $s_a \geq 1$

Substituting this back into the definition (*above*) for the covariance (σ_{yz}), yields:

$$\sigma_{yz} = \{(1/s_a) - E(y)\} * E(z)$$

or with rearrangement: $1/s_a = E(y) + \sigma_{yz}/E(z) \leq 1$

Also, in this case: $P(MS, E \,|\, G, S_E) = m * E(yz) = m * E(z)/s_a = P(MS, E \,|\, G, DZ_E)/s_a$

or: $s_a = P(MS \,|\, G, DZ_E)/P(MS \,|\, G, S_E) \geq 1$

We define the term (IG_E) in an analogous manner to (MZ_E) – *see above*. Moreover, because everyone who develops MS must experience the event (E), because both *MZ*- and *DZ*-twins disproportionately share the (E_{twn}) and (E_{sib}) environments with their co-twin, and because siblings (including twins) disproportionately

share their (E_{sib}) environment with their co-sibling(s), and because everyone equally shares their (E_{pop}) environment, therefore, with respect to the environmental experiences of the proband, it will be the case that:

$$P(E \mid MZ_E) = P(E \mid MZ_{MS}) = P(E \mid G, DZ_{MS}) = P(E \mid G, DZ_E)$$

Moreover, from the definition of (IG_{MS}) and because the (E_{sib}) environment doesn't seem to contribute to the event (MS, E) and because genotype is independent of (E_T), then, in this circumstance:

$$P(E \mid G, S_E) = P(E \mid G, S_{MS}) = P(E \mid IG_{MS}) = P(E \mid IG_E) = P(E \mid G)$$

Moreover: $P(MS \mid DZ_{MS}, E_T) = P(MS, G \mid DZ_{MS}, E_T) = P(MS \mid G, DZ_{MS}, E_T) * P(G \mid DZ_{MS})$

and: $P(MS \mid S_{MS}, E_T) = P(MS \mid G, S_{MS}, E_T) * P(G \mid S_{MS})$

where: $P(G \mid DZ_{MS}) = P(G \mid S_{MS})$

Therefore, to convert $P(MS \mid MZ_{MS}, E_T)$ into $P(MS \mid IG_{MS}, E_T)$, we can remove the impact of the shared (E_{twn}) environment of *MZ*-twins, during (E_T), using observable population parameters such that:

$$s_a = P(MS \mid DZ_{MS}, E_T)/P(MS \mid S_{MS}, E_T)$$

and, thus: $P(MS \mid IG_{MS}, E_T) = P(MS \mid MZ_{MS}, E_T)/s_a$

{NB: In this development of the adjustment factor (s_a), we have assumed that the degree of sharing the (E_{twn}) environment is the same for MZ- and DZ-twins. We have also assumed, based on evidence [10-16], that the (E_{sib}) environment doesn't impact the likelihood of a person subsequently developing MS. However, either one (or both) of these assumptions may be incorrect. For example, certain (E_{twn}) exposures may be more or less likely to be shared among MZ-twins compared to DZ-twins, in which case our estimate for (s_a) would be either an under or an overestimate. Nevertheless, if either the (E_{twn}) or the (E_{sib}) environments are important to MS pathogenesis, then some adjustment is necessary. It is for this reason that, in our analysis, we consider a wide range of possible values for (s_a), including those circumstances under which no adjustment is necessary – see below.}

1c. Adjustment for the Susceptible Women and Men Considered Together

Assertion: $P(MS \mid IG_{MS}) = 0.136$

Proof: We will here use the point-estimates from the Canadian twin study [7] of:

$$P(MS \mid MZ_{MS}) = 0.253$$
$$P(MS \mid DZ_{MS}) = 0.054$$
$$P(MS \mid S_{MS}) = 0.029$$

Following *Sections 1a–b* (*above*), we can derive a point estimate for the value of $\{P(MS \mid IG_{MS})\}$ as:

$$s_a = P(MS \mid DZ_{MS})/P(MS \mid S_{MS}) = 0.054/0.029 = 1.86$$

$$P(MS \mid IG_{MS}) = P(MS \mid MZ_{MS})/s_a = 0.253/1.86 = 0.136$$

1d. Adjustments for Susceptible Women and Men Considered Separately

Assertions: $P(MS \mid F, IG_{MS}) = P(MS \mid F, MZ_{MS})/1.86$

$P(MS \mid M, IG_{MS}) = P(MS \mid M, MZ_{MS})/1.88$

Proof: We define two parameters ($e \geq 1$) and ($f \geq 1$) such that:

$$P(MS \mid F, IG_{MS}) = P(MS \mid F, MZ_{MS})/e$$

$$P(MS \mid M, IG_{MS}) = P(MS \mid M, MZ_{MS})/f$$

Again, we will use the point-estimates from the Canadian twin study [7] of:

$$P(F \mid MS) = P(F \mid IG_{MS}) = 0.685$$

$$P(F \mid MS, MZ_{MS}) = P(F \mid MS, IG_{MS}) = 0.92$$

$$P(MS \mid F, MZ_{MS}) = 0.34$$

$$P(MS \mid M, MZ_{MS}) = 0.065$$

We can deconstruct the term $\{P(MS, F \mid IG_{MS})\}$ in two different ways:

$$P(MS, F \mid IG_{MS}) = P(F \mid IG_{MS}) * P(MS \mid F, IG_{MS}) = (0.685 * 0.34)/e$$

and: $$P(MS, F \mid IG_{MS}) = P(MS \mid IG_{MS}) * P(F \mid MS, IG_{MS}) = (0.136 * 0.92)$$

Combining these two equations leads to:

$$e = (0.685 * 0.34)/(0.136 * 0.92) = 1.86$$

Similarly: $$P(MS, M \mid IG_{MS}) = P(M \mid IG_{MS}) * P(MS \mid M, IG_{MS}) = (0.315 * 0.065)/f$$

and: $$P(MS, M \mid IG_{MS}) = P(MS \mid IG_{MS}) * P(M \mid MS, IG_{MS}) = (0.136 * 0.08)$$

leading to: $$f = (0.315 * 0.065)/(0.136 * 0.08) = 1.88$$

Thus, the point estimate for the impact of the shared (E_{twn}) environment on the likelihood that a proband *MZ*-twin has or will develop MS, given the fact that their co-twin also has or will develop MS, is essentially identical for both *women* and *men*.

2. *Enrichment of Genotypes*

2a. Enrichment from the subset $(\boldsymbol{G})$ *to the subset* $(\boldsymbol{MS})$

Assertion: It can be demonstrated that more penetrant genotypes are "*enriched*" in the (G, MS), (F, G, MS) and (M, G, MS) subsets compared, respectively, to the subsets of susceptible individuals (G), (F, G) and (M, G).

Proof: During any *Time Period*, we consider the two ratios, (A) and (B), such that:

$$A = P(MS \mid F, G)/P(MS \mid G) = x_1/x$$

and: $$B = P(MS \mid M, G)/P(MS \mid G) = x_2/x$$

where: $$P(MS \mid G) = P(MS, F \mid G) + P(MS, M \mid G)$$

Therefore, during any *Time Period*, the following two relationships must hold simultaneously:

$$1 = P(F \mid G) + P(M \mid G)$$

and: $$1 = A * P(F \mid G) + B * P(M \mid G)$$

Consequently, during any *Time Period*, it is clear that:

$$A = 1 \quad if\ and\ only\ if: \quad B = 1$$

and: $$A > 1 \quad if\ and\ only\ if: \quad B < 1$$ (and *vice versa*)

Moreover: $$P(F \mid MS, G) = P(F, G) * P(MS \mid F, G)/P(MS, G) = A * P(F \mid G)$$

and similarly: $$P(M \mid MS, G) = B * P(M \mid G)$$

Therefore, if $(A < 1 < B)$ – i.e., if *men* are more penetrant than *women* – then:

$$P(F \mid MS) < P(F \mid G) \quad \& \quad P(M \mid MS) > P(M \mid G)$$

Conversely, if $(A > 1 > B)$ – i.e., if *women* are more penetrant than *men* – then:

$$P(F \mid MS) > P(F \mid G) \quad \& \quad P(M \mid MS) < P(M \mid G)$$

In either case, the more penetrant gender will be described as being "*enriched*" in the (MS) subset in comparison to the (G) subset.

In addition, considered separately, and partitioning each of the subsets (F, G) and (F, G) into their high- and low-penetrance sub-subsets, $(G1')$ and $(G2')$, respectively – *see Methods #1A; Main Text* – an analogous argument demonstrates that an "*enrichment*" of more penetrant genotypes will also occur within each of these two sub-subsets such that:

$$P(F, G1' \mid MS > P(F, G1' \mid G)$$

and: $$P(M, G1' \mid MS) > P(M, G1' \mid G)$$

2b. Enrichment from the subset $(\boldsymbol{G}, \boldsymbol{MS})$ to the subset $(\boldsymbol{G}, \boldsymbol{MS}, \boldsymbol{MZ_{MS}})$

Assertion: A similar "*enrichment*" of more penetrant genotypes will also occur in the (G, MS, MZ_{MS}), (F, G, MS, MZ_{MS}) and (M, G, MS, MZ_{MS}) subsets compared, respectively, to the subsets of susceptible individuals (G, MS), (F, G, MS) and (M, G, MS).

Proof: Following the same logic as that, which is used for the demonstration of *Assertion 2a* (*above*), this assertion has been demonstrated previously [3].

2c. Current Enrichment of Women Compared to Men

Assumption: $Zw_2 = P(MS \mid F, G)_2 > P(MS \mid M, G)_2 = Zm_2$

Argument: *Currently*, the evidence strongly favors of an arrangement (*above*) in which: $(A > 1)$ or, equivalently, in which: $(Zw_2 > Zm_2)$. First, the (*F:M*) *sex ratio* has increased by a similar amount (i.e., at a similar rate) between every two *Time Periods* in the Canadian data except one [6]. Thus, the observed increase in MS prevalence over time has disproportionately impacted the prevalence in *women* [6,22-30]. Indeed, from *Equation 1b* (*Main Text*), during the "*current*" *Time Period*, it must be that:

$$(F{:}M)_2\ sex\ ratio = \left\{\frac{p}{1-p}\right\} * \left(\frac{Zw_2}{Zm_2}\right) = \frac{p'_2}{(1-p'_2)} \qquad (\textit{Equation S1a})$$

The value of (p) is unknown but fixed, regardless of *Time Period*, and the values of (Zw_2) and (Zm_2) are also unknown but fixed during the *current Time Period* (*see Section 6a, below*). Considering *Equation S1a*, an increasing *(F:M) sex ratio* with increasing exposure can only be explained by the penetrance in susceptible *women* (Zw) increasing at a faster rate compared to the rate in susceptible *men* (Zm). If the penetrance of MS in susceptible *women* was changing at the same rate as in susceptible *men* (whether increasing, decreasing, or remaining constant), the (*F:M*) *sex ratio*, would stay constant. In contrast to the fixed value of (p), the value of (p') depends upon the *Time Period* and it is an observed parameter. Thus, the only possible circumstances under which: $(Zm_2 \geq Zw_2)$, are also those in which the value of: (p) is greater than (or equal to) the "*current*" value for (p'_2). From our parameter estimates (*see Methods #2A; Main Text*), the acceptable range for the parameter (p'_2) is such that:

$$0.74 \leq p'_2 \leq 0.78$$

Also, because, as discussed in *Section 6c* (*see below*), the limiting values for the exponential response curves in susceptible *men* and *women* are related such that, with increasing exposure, the ratio (Zw/Zm) will always approach the limit of $(\boldsymbol{d}/\boldsymbol{c})$. Thus:

$$\lim_{a\to\infty}(Zw/Zm) = \boldsymbol{d}/\boldsymbol{c} \qquad (\textit{Equation S1b})$$

If circumstances were such that: $(\boldsymbol{c} = \boldsymbol{d} \leq 1)$ then: $(Zw_2/Zm_2 < 1)$. When the limit $(\boldsymbol{d}/\boldsymbol{c})$ is reached, it will be the case that: $(Zw = Zm \leq 1)$; so that the (*F:M*) *sex ratio* will steadily increase until it reaches the limiting value of:

$$(F\!:M)\ sex\ ratio = \left\{\frac{p}{1-p}\right\}$$

However, the observed "*current*" value of $(p'_2 = 0.76)$ in Canada [6] is greater than the proportion of *women* among MS patients observed during every previous *Time Period* represented by the Canadian *population* data [6] and, also, greater than that for every observation made of MS since Charcot's initial clinical description in the 19th century [3,40,76-79]. In such a circumstance, we would have to conclude, therefore, that *men*, throughout the history of MS, have had a consistently greater penetrance than *women* but that *women* are considerably more likely than *men* to be genetically susceptible. Such a conclusion is counter intuitive. In addition, there is an inherent tension between the conclusion that the penetrance of MS is increasing faster in *women* than *men* (which suggests that: $R > 1$) and the conclusion that $(Zm > Zw)$ throughout the history of MS (which suggests that: $R < 1$) – *see Section 6g; below*.

Second, although the current *relative penetrance* of MS for the sets (F, G) and (M, G), is not an observed parameter, nevertheless, the *penetrance* values for the sets (F, G, MZ_{MS}) and: (M, G, MZ_{MS}) are directly observable and, currently [6], this *penetrance* is (5.7)-fold higher for *women* than it is for *men*.

$$\text{Thus:} \quad 0.34 = P(MS \,|\, F, G, MZ_{MS})_2 \gg P(MS \,|\, M, G, MZ_{MS})_2 = 0.065$$

This difference in penetrance between genders is highly significant such that:

$$\chi^2 = 10.5 \quad ; \quad p = 0.001$$

As a consequence of this penetrance difference, and considering *MZ*-twins who either are of unknown concordance or are known to be concordant – i.e., who, respectively, are members of either the (MZ_{MS}) subset or the (MS, MZ_{MS}) sub-subset – this study [7] indicates that *women* are being continuously *enriched* such that:

$$P(F) = 0.5 < (F \,|\, MZ_{MS}) = 0.685 < P(F \,|\, MS, MZ_{MS}) = 0.92$$

Because any proband *MZ*-twin is genetically "identical" to their (MZ_{MS}) co-twin, the probands of such co-twins are already "*enriched*" for more penetrant genotypes compared to the general population (*see above*). Consequently, extrapolating from the fact that this "*enriched*" group of *women* has a considerably greater penetrance than similarly "*enriched*", strongly suggests that, *currently*, the *relative penetrance* is such that susceptible *women* are more *penetrant* than susceptible *men* (*see Sections 2a–b; above*). Nevertheless, there are two circumstances, which might, potentially, make any such extrapolation inaccurate. First, *MZ*-twins share their (E_{twn}) and (E_{sib}) environments, which might cause an increase in the *penetrance* relative to the same genotypes under non-twin conditions [4]. If the impact of these environments were markedly disproportionate for the different two genders this could change the *relative penetrance*. However, this concern is probably unwarranted because, observationally, the environmental impact of sharing these environments is

the same for both *men* and *women* (*see Section 1d; above*) and, thus, this circumstance won't impact the *relative penetrance*. Second, as noted *above*, there will be an enrichment of more penetrant genotypes that occurs in the probands of (MZ_{MS}) co-twins compared to non-twin conditions. If this enrichment were markedly disproportionate between the genders, then, potentially, this could also impact the *relative penetrance*. However, for this to explain the large observed discrepancy in the *MZ*-twin concordance rates for *men* and *women* (*see above*), would require an extreme difference in the variance of individual penetrance values between the (F, G) subset and the (M, G) subset [3,4] – i.e., $(\sigma_{X_1}^2 \gg \sigma_{X_2}^2)$; or, equivalently: $(r \gg s)$ – *see Section 3a; Equations S2f–i & S3a–d (below)*. This seems improbable. Therefore, without making any assertions regarding the circumstances that pertain during earlier *Time Periods*, we assume that, currently, the *penetrance* in *women* is greater than the *penetrance* in *men* so that:

$$Zw_2 = P(MS \mid F, G)_2 > P(MS \mid M, G)_2 = Zm_2$$

and, thus, from *Equation S1a* (*above*): $p < p_2'$

{NB: If our assumption that, currently, $(Zw_2 > Zm_2)$*, were incorrect, then, as noted, up until the present time, susceptible men would always have had a greater penetrance than susceptible women. Moreover, this would also indicate that men comprise only a small proportion* $(\leq 26\%)$ *of all susceptible individuals or, equivalently, that:* $\{P(G \mid M) \leq 0.36)\}$ *and that:* $\{P(G) \leq 0.68)\}$*. Nevertheless, in such a case, conditions similar to those depicted in* $Figure\ 1C$ *(although approaching a sex ratio greater than unity) might be possible. However, using the definitions from the Longitudinal Model (see Section 4a; below and Table 2; Main Text), and using the substitution analysis (see Methods 2C; Main Text) for those conditions where* $(p \geq 0.74)$*, we found that every solution was restricted such that:*

$$P(MS \mid F, G)_2 = P(MS \mid M, G)_2 \ \ and: \ \ p = p'_2$$

$$P(MS \mid F, G)_1 < P(MS \mid M, G)_1 \ \ and: \ \ p > p'_1$$

$$R^{app} > 1 \ ; \ r > 2; \ \ r/s \geq 2 \ ; \ \ and: \ P(G) < 0.25$$

Notably, if the hazards are proportional, any solution for which $(p = p'_2)$ *implies that the response curves in both women and men have currently (i.e., by Time Period #2) reached their maximum value limits (see Section 6c; Equation S12d) where:* $(Zw = Zm)$*. This circumstance further implies that no further increase in the (F:M) sex ratio is possible. For our current Time Period to have precisely coincided with this point in the response curve seems surprising, especially given that the observed sex ratio has been steadily increasing up to this point, without any suggestion of tapering off [6].*

Also, as it pertains to other aspects of either Model, it is worth noting that, because: $\{P(F) = 0.5\}$, *any circumstance for which:* $(p \neq 0.5)$, *requires that:* $[P(G) < 1]$ *and, therefore, would indicate that MS is a "genetic" disease in the sense discussed in Methods #1E (Main Text).}*

3. ***Cross-sectional Model***

3a. Model Development

For notational simplicity, we define (*see Table 2; Main Text*): $p = P(F \mid \mathrm{G})$; $x = P(MS \mid G)_2$; $x' = P(MS \mid IG_{MS})_2$; $x_1 = P(MS \mid F, G)_2$; $x_1' = P(MS \mid F, IG_{MS})_2$; $x_2 = P(MS \mid M, G)_2$; $x_2' = P(MS \mid M, IG_{MS})_2$; and the ratios: $r = x_1'/x_1$; and: $s = x_2'/x_2$;

Assertions: **A.** $\forall G_i \in G: \quad P(G_i, MS \mid MZ) = P(G_i, MS)$

$\forall G_i \in G: \quad P(G_i \mid IG_{MS}) = P(G_i \mid MZ_{MS}) = P(G_i \mid MS)$

$P(MZ_{MS}) = P(MS)$

$P(F \mid G, IG_{MS}) = P(F \mid G, MZ_{MS}) = P(F \mid G, MS) = P(F \mid MS)$

B. $P(IG_{MS}) = P(MZ_{MS}) = P(MS)$

C. $x = (x'/2) \pm \sqrt{(x'/2)^2 - \sigma_X^2}$

D. $0 \leq \sigma_X^2 \leq (x'/2)^2$

$\sigma_X^2 = x(x' - x)$

Definitions and Assumptions: The subsets (G) and (G^c) have already been defined *(see Methods #1A; Main Text*) and as noted there:

$$\forall\ G_i \in G: \quad x_i = P(MS \mid G_i)$$

Thus, (x_i) represents the MS-penetrance for the *i*[th] individual in the (G) subset during <u>any</u> specific *Time Period* and it is unique to the *i*[th] individual, regardless of whether it is identical (in value) to that of another individual. Notably, by definition (*see Methods #1A; Main Text*), all members of the subsets (MS), and (IG_{MS}) are also members of the (G) subset, so that, both:

$$(MS) = (G, MS) \quad \text{and:} \quad (IG_{MS}) = (G, IG_{MS})$$

Also, as discussed in *Methods #1A; Main Text*, we defined the set (X) to include the penetrance value for each of the (m) members of the (G) subset and its variance to be (σ_X^2). Thus, we define this set such that: $X = \{x_i\}$; where: $(i = 1,2, \dots, m)$; and: $Var(X) = \sigma_X^2$. Letting (x_G) be a random variable representing any of the $\{x_i\}$ elements within the set (X), and because all genotypes in a population are unique, it follows that:

$$P(G) = m/N$$

$$\forall G_i \in G: \ P(G_i \mid G) = \frac{P(G_i)}{P(G)} = \frac{(1/N)}{(m/N)} = 1/m$$

$$E(x_G) = \sum_{i=1}^{m}(x_i) * (1/m) = P(MS \mid G) = x \qquad (\textit{Equation S2a})$$

$$E(x_G^2) = \sum_{i=1}^{m}(x_i^2) * (1/m) = x^2 + \sigma_X^2 \qquad (\textit{Equation S2b})$$

$$x' = P(MS \mid G, IG_{MS}) = \sum_{i=1}^{m} P(MS, G_i \mid G, IG_{MS}) \qquad (\textit{Equation S2c})$$

As noted earlier, if $\{\sigma_X^2 \neq 0\}$, then the subset (G) can be partitioned into two mutually exclusive subsets, $(G1)$ and $(G2)$, suitably defined, such that: $(x_1 > x_2)$

We now specify our two assumptions:

Assumption #1

Because *MZ*-twinning is generally thought to be non-hereditary [18-20], we assume that every person (i.e., genotype) in the general population (Z) has the same chance, *a priori*, of having an *MZ*-twin (i.e., *MZ*-status is independent of genotype). Thus, we assume that, for any *Time Period*:

$$1.\ \forall G_k \in Z:\ P(MZ \,|\, G_k) = P(MZ)$$

Because this relationship applies to every genotype in (*Z*), therefore, also:

$$2.\ \forall G_i \in G:\ P(MZ \,|\, G_i) = P(MZ)$$

If, rarely, *MZ*-twinning were familial [19], it would still be the case that:

$$3.\ \forall G_k \in Z:\ P\big(MZ \,|\, G_k\big) \approx P(MZ)$$

Finally, even if some cases of *MZ*-twinning were thought to be familial [19], the exact relationship (*#2, above*) still follows if those genetic factors, which are associated with *MZ*-twining, are independent of those genetic factors associated with MS. Here we assume that either this or #1 (*above*) pertains.

Assumption #2

The penetrance of MS for a proband *MZ*-twin, whose co-twin is of unknown status, is assumed to be the same as if that genotype had occurred without having an *MZ* co-twin (i.e., the penetrance of MS for each genotype is independent of *MZ*-status). This assumption translates to assuming that the impact of experiencing any particular (E_{twn}) and (E_{sib}) environments together with an *MZ* co-twin is the same as the impact of experiencing the same (E_{twn}) and (E_{sib}) environments alone. Alternatively, it translates to the testable hypothesis that the mere fact of having an *MZ* co-twin does not alter the (E_{twn}) and (E_{sib}) environments in such a way that MS becomes more or less likely in both twins. Thus, we are here assuming that, for any *Time Period*:

$$\forall G_i \in G:\ P(MS \,|\, G_i, MZ) = P(MS \,|\, G_i)$$

Proof of Assertion A:

From *Assumption #1*, it follows that:

$$\forall G_k \in Z:\quad P(G_k, MZ) = P(G_k) * P(MZ \,|\, G_k) = P(G_k) * P(MZ)$$

and therefore: $\quad \forall G_k \in Z:\ P\big(G_k \,|\, MZ\big) = P(G_k, MZ)/P(MZ) = P(G_k)$

Consequently, also: $\quad \forall G_i \in G:\ P\big(G_i \,|\, MZ\big) = P(G_i)$

From this conclusion, from the definitions relating to (MZ_{MS}) – *see Methods #1C; Main Text* – and from *Assumption #2*, it follows that, for any *Time Period*:

$$\forall G_i \in G\text{:}\ P(G_i, MZ_{MS}) = P\big(MS, G_i \,\big|\, MZ\big) = P\big(G_i \,\big|\, MZ\big) * P\big(MS \,\big|\, G_i, MZ\big)$$

$$= P(G_i) * P(MS \,|\, G_i) = P(MS, G_i)$$

and: $$P(MZ_{MS}) = P(MS \,|\, MZ) = \textstyle\sum_{i=1}^{m} P(\,G_i \,|\, MZ) * P(MS \,|\, G_i, MZ)$$

$$= \textstyle\sum_{i=1}^{m} P(G_i) * P(MS \,|\, G_i) = P(MS)$$

This last conclusion is also evident from the definition of (MZ_{MS}) – see *Methods #1C; Main Text*. From these two equivalences, and from the definition of (IG_{MS}) – *see; Sections 1a–c; above; see also Methods #1D; Main Text* – we conclude that, for any *Time Period*:

$$\forall G_i \in G\text{:}\ \ P\big(G_i \,\big|\, IG_{MS}\big) = P\big(G_i \,\big|\, MZ_{MS}\big) = \frac{P(G_i, MZ_{MS})}{P(MZ_{MS})} = \frac{P(G_i, MS)}{P(MS)} = P\big(G_i \,\big|\, MS\big)$$

Therefore, also:

$$P(F \,|\, IG_{MS}) = \textstyle\sum_{d=1}^{mp} P(G_{dw^s} \,|\, IG_{MS}) = \sum_{d=1}^{mp} P(G_{dw^s} \,|\, MS) = P(F \,|\, MS)$$

Thus: $$P(F \,|\, G, IG_{MS}) = P(F \,|\, G, MS) = P(F \,|\, MS)$$

and similarly: $$P(M \,|\, G, IG_{MS}) = P(M \,|\, G, MS) = P(M \,|\, MS)$$

Proof of Assertion B:

From the definition of (IG_{MS}) – *see Section 1a* (*above*)*; see also Methods #1D; Main Text* – and from *Assertion A*, it directly follows that, for any *Time Period*:

$$P(IG_{MS}) = P(MZ_{MS}) = P(MS)$$

Proof of Assertion C:

From the definitions of (G) & (IG_{MS}) – *see Section 1a* (*above*)*; see also Methods #1A & 1D; Main Text* – it directly follows that:

$$P(MS \,|\, G_i, IG_{MS}) = P(MS \,|\, G_i, G, IG_{MS}) = P(MS \,|\, G_i, G) = P(MS \,|\, G_i) = x_i$$

Consequently, during any *Time Period*, the probability $P(MS, G_i \,|\, G, IG_{MS})$ can be re-expressed as:

1. $P(MS, G_i \,|\, G, IG_{MS}) = P(G_i \,|\, G, IG_{MS}) * P(MS \,|\, G_i, G, IG_{MS})$

$$= P(G_i \,|\, G, IG_{MS}) * (x_i)$$

From *Assertion A* and from the definitions of (G) & (IG_{MS}) – *see Sections 1a* (*above*)*; see also Methods #1A & 1D; Main Text* – the term $P(G_i \,|\, G, IG_{MS})$ can be re-expressed as:

$$2.\quad P(G_i \,|\, G, IG_{MS}) = P(G_i \,|\, G, MS) = P(G_i, G, MS)/P(MS, G)$$
$$= P(MS \,|\, G_i, G) * P(G_i, G)/P(MS, G)$$
$$= (x_i) * P(G_i \,|\, G)/P(MS \,|\, G) = (x_i) * (1/m)/x$$

Combining *1 & 2 (above*) yields:

$$P(MS, G_i \,|\, G, IG_{MS}) = (x_i)^2 * (1/m)/x$$

However, from *Equation S2c*, it is the case that:

$$x' = P(MS, G \,|\, G, IG_{MS}) = \sum_{i=1}^{m} P(MS, G_i \,|\, G, IG_{MS})$$

where: $\sum_{i=1}^{m} P(MS, G_i \,|\, G, IG_{MS}) = \sum_{i=1}^{m} \left(x_i^2\right) * (1/m)/x = E(x_G^2)/x$

Therefore, from *Equation S2b*, it follows that:

$$x' = (x^2 + \sigma_X^2)/x = x + \sigma_X^2/x \qquad (\textit{Equation S2d})$$

Equation S2d can be rearranged to yield a quadratic equation in (*x*) such that:

$$x^2 - (x')x + \sigma_X^2 = 0$$

This quadratic can be solved to yield:

$$x = (x'/2) \pm \sqrt{(x'/2)^2 - \sigma_X^2} \qquad (\textit{Equation S2e})$$

Defining the variance of penetrance values within the subsets of susceptible *women* and *men* as $(\sigma_{X_1}^2)$ and $(\sigma_{X_2}^2)$, respectively, the same line of argument also leads to the conclusions that:

$$x_1^2 - (x_1')x_1 + \sigma_{X_1}^2 = 0 \qquad (\textit{Equation S2f})$$

$$x_2^2 - (x_2')x_2 + \sigma_{X_2}^2 = 0 \qquad (\textit{Equation S2g})$$

$$x_1 = (x_1'/2) \pm \sqrt{(x_1'/2)^2 - \sigma_{X_1}^2} \qquad (\textit{Equation S2h})$$

and: $$x_2 = (x_2'/2) \pm \sqrt{(x_2'/2)^2 - \sigma_{X_2}^2} \qquad (\textit{Equation S2i})$$

Proof of Assertion D:

Equation S2e has real solutions only for the range of:

$$0 \le \sigma_X^2 \le (x'/2)^2 \qquad (\textit{Equation S3a})$$

and, also, from *Equations S2f–g*:

$$0 \le \sigma_{X_1}^2 \le (x_1'/2)^2 \quad \text{and:} \quad 0 \le \sigma_{X_2}^2 \le (x_2'/2)^2 \qquad (\textit{Equations S3b})$$

Notably, the maximum variance (σ^2) for any distribution [38] on the closed interval $[a, b]$ is:

$$\sigma^2 = \{(b-a)/2\}^2$$

Therefore, irrespective of any *Assumptions* we have made (*see above*), the variances ranges provided in *Equations S3a–b* are the maximum possible variance ranges for *any* distribution on each of the respective closed intervals $[0, x']$, $[0, x_1']$ and: $[0, x_2']$.

Also, *Equation S2d* can be re-arranged to yield:

$$\sigma_X^2 = x(x' - x) \qquad (Equation\ S3c)$$

and, similarly: $$\sigma_{X_1}^2 = x_1(x_1' - x_1) \qquad (Equation\ S3d)$$

and: $$\sigma_{X_2}^2 = x_2(x_2' - x_2) \qquad (Equation\ S3e)$$

3b. Assertion C Solutions

Equation S2e has two solutions – the so-called *Upper Solution* and the *Lower Solution*, depending upon the value of the (±) sign. The *Upper Solution* represents the gradual transition from a distribution, when $(\sigma_X^2 = 0)$, in which everyone has a penetrance of (x') to a bimodal distribution, when $\{\sigma_X^2 = (x'/2)^2\}$, in which half of the (G) subset has a penetrance of (x') and the other half has a penetrance of zero. Although, under *some* environmental conditions: $(\forall\, x_i \in X{:}\ \ x_i > 0)$, as noted previously (*see Methods #1A, Main Text*), there may be certain environmental conditions, in which, for some individuals in the (G) subset:

$$P(MS \mid G_i, E_T) = 0$$

Therefore, the *Upper Solution*, during any particular *Time Period*, is constrained such that:

$$x'/2 \le x \le x'$$

The *Lower Solution* represents the gradual transition from the bimodal distribution described above to increasingly extreme and asymmetric distributions [3]. The *Lower Solution*, however, is further constrained by the requirement of *Equation S2d* that when: $(\sigma_X^2 = 0)$ then: $(x = x')$. Therefore, the *Lower Solution* is constrained such that:

$$0 < x \le x'/2$$

3c. Quadratic Solutions for Women and Men

Moreover, regardless of whether the *Upper* or *Lower Solution* pertains, the values that (x_1) and (x_2) can take are further constrained.

Assertions:

1. $$x_1 = Zw = \frac{x + \sqrt{x^2 - \{1 + (r/s)(1-p)/p)\}\{x^2 - xx'(1-p)/s\}}}{p + (r/s)(1-p)}$$

2. $$x_2 = Zm = \frac{x - \sqrt{x^2 - \{1 + (s/r)(p/(1-p))\}\{x^2 - xx'\,p/r\}}}{(1-p) + (s/r)p}$$

Proof: Restating *Equation 1c* (*Main Text*):

$$x = px_1 + (1-p)x_2$$

with re-arrangement, this becomes:

$$x_2 = [x - p(x_1)]/(1-p) \qquad (\textit{Equation S4a})$$

Also: $x' = P(MS \mid G, IG_{MS}) = P(MS, F \mid G, IG_{MS}) + P(MS, M \mid G, IG_{MS})$

Therefore, from *Assertion 3A* (*above*):

$$P(MS, F \mid G, IG_{MS}) = P(F \mid G, IG_{MS}) * x_1' = P(F \mid G, MS) * x_1'$$

and: $P(MS, M \mid G, IG_{MS}) = P(M \mid G, IG_{MS}) * x_2' = P(M \mid G, MS) * x_2'$

where: $P(F \mid G, MS) = P(F, MS \mid G)/P(MS \mid G) = p(x_1)/x$

and, similarly: $P(M \mid G, MS) = (1-p)(x_2)/x$

so that: $xx' = p(x_1)(x_1') + (1-p)(x_2)(x_2') = pr * (x_1)^2 + (1-p)s * (x_2)^2$

or: $(x_2)^2 = [xx' - pr(x_1)^2]/[(1-p)s]$ (*Equation S4b*)

Consequently, we have two different estimates for $(x_2)^2$ – i.e., *Equations S4a and S4b, above*.

Combining these two estimates {to eliminate the (x_2) parameter} yields:

$$[\{x - p(x_1)\}/(1-p)]^2 = (x_2)^2 = [xx' - pr(x_1)^2]/[(1-p)s]$$

or: $\{x - p(x_1)\}^2 = \{xx' - pr(x_1)^2\}(1-p)/s = \{xx'(1-p)/s\} - (r/s)p(1-p)(x_1)^2$

and, finally: $x^2 - 2xp(x_1) + p^2(x_1)^2 - xx'(1-p)/s + (r/s)p(1-p)(x_1)^2 = 0$

Rearrangement, yields a quadratic equation in (x_1) such that:

$$\{p^2 + (r/s)p(1-p)\}(x_1)^2 - \{2xp\}(x_1) + \{x^2 - xx'(1-p)/s\} = 0$$

Because of the definition that: $(x_1 > x_2)$ – *see Section 3a, above* – this is solved for (x_1) as:

$$x_1 = Zw = \frac{x + \sqrt{x^2 - \{1 + (r/s)(1-p)/p)\}\{x^2 - xx'(1-p)/s\}}}{p + (r/s)(1-p)} \qquad (\textit{Equation S5a})$$

Equation S4a (*above*) can then be solved for (x_2). Alternatively, reframing the *above* argument to eliminate the (x_1) parameter instead of (x_2), yields:

$$x_2 = Zm = \frac{x - \sqrt{x^2 - \{1 + (s/r)(p/(1-p)\}\{x^2 - xx'\,p/r\}}}{(1-p) + (s/r)p} \qquad (\textit{Equation S5b})$$

4. Longitudinal Model:

4a. Model Development

Following standard survival analysis methods [39], we define the cumulative survival $\{S(u)\}$ and failure $\{F(u)\}$ functions where: $F(u) = 1 - S(u)$. These functions are defined separately for *men* $\{S_m(u)\ and\ F_m(u)\}$ and for *women* $\{S_w(u)\ and\ F_w(u)\}$. In addition, we define the hazard-rate functions for developing MS at different exposure-levels (u) in susceptible *men* and *women* {i.e., $h(u)$ and $k(u)$, respectively}. These hazard-rate functions for *women* and *men* may or may not be proportional to each other but, if they are proportional, then: $k(u) = R * h(u)$, where $(R > 0)$ represents the hazard proportionality factor. Furthermore, as defined previously (*see Methods #1B; Main Text*), the term $P(E \mid G, E_T)$ represents the probability of the event that a member of the (G) subset, selected at random, experiences an environmental exposure "*sufficient*" to cause MS in them, given the prevailing environmental conditions of the time (E_T). We define the exposure (u) as the odds of this event during the *Time Period* (E_T) such that:

$$u = P(E \mid G, E_T)/[1 - P(E \mid G, E_T)]$$

We further define $H(a)$ to be the cumulative hazard function (for *men*) at an exposure-level $(u = a)$ such that:

$$H(a) = \int_0^a h(u)du$$

Similarly, we define $K(a)$ to be the cumulative hazard function (for *women*) at the same exposure-level of $(u = a)$ such that:

$$K(a) = \int_0^a k(u)du$$

And, if the hazards are proportional, then:

$$K(a) = \int_0^a R * h(u)du = R * H(a)$$

For *men*, following the usual definition of the hazard function [39] that:

$$h(u) = f_m(u)/S_m(u)$$

together with the fact that, by definition:

$$f_m(u) = d[F_m(u)]/du = -d[S_m(u)]/du$$

a standard derivation from survival analysis methods [39] demonstrates that, for *men*, because:

$$h(u)du = -d[S_m(u)]/S_m(u)$$

Therefore, we can re-express the cumulative hazard function $\{H(a)\}$ such that:

$$H(a) = -\int_0^a d[S_m(u)]/S_m(u) = \ln[S_m(0)] - \ln[S_m(a)]$$

and: $$H(0) = \ln[S_m(0)] - \ln[S_m(0)] = 0$$

Because we are measuring exposure as the odds that a member of the (G) subset receives an environmental exposure "*sufficient*" to cause MS, given the environmental conditions at the time, by definition, when: $\{P(E \mid G, E_T) = 0\}$, no member of (G) can develop MS {i.e., $S_m(0) = 1\}$. In this circumstance: $\{\ln[S_m(0)] = \ln(1) = 0\}$; and therefore:

$$S_m(a) = e^{-H(a)}$$

Consequently, the cumulative survival function is exponentially related to the integral of the underlying hazard function – i.e., the cumulative hazard function. Even in the unlikely circumstance that the hazard function is discontinuous at some points, the function will still be integrable in all realistic scenarios. In this circumstance, the failure function for susceptible *men* becomes:

$$F_m(a) = 1 - S_m(a) = 1 - e^{-H(a)}$$

{NB: In this circumstance, we are using the cumulative hazard function, $H(a)$, as a measure of exposure for susceptible men, not as a measure of either survival or failure. By contrast, failure, as defined here, is the event that a person develops MS during their lifetime. The term: $Zm = P(MS, E \mid M, G, E_T)$ represents the probability that, during the Time Period (E_T), this failure event occurs for a randomly selected individual from the (M, G) subset of (Z). Notably, also, the exposure-measure of $H(a)$ is being used in preference to the, perhaps, more intuitive measure of exposure $(u = a)$ provided above. Nevertheless, when $\{P(E \mid G, E_T) = 0\}$; both exposure measures are zero – i.e., $\{a = 0\}$ and $\{H(a) = 0\}$. Also, as the value of: $\{P(E \mid G, E_T) \to 1\}$; both exposure measures become infinite: i.e., $\{a \to \infty\}$ and $\{H(a) \to \infty\}$. And, finally, both exposure measures increase monotonically with increasing $P(E \mid G, E_T)$. Therefore, the mapping of the $(u = a)$ measure to the $H(a)$ measure is both one-to-one and onto. Consequently, these two measures of exposure are equivalent, and the use of either exposure scale is appropriate. Although the relationship of the $H(a)$ scale to $P(E \mid G, E_T)$ is less obvious than it is for the $(u = a)$ scale where: $P(E \mid G, E_T) = a/(a + 1)$, the $H(a)$ scale, nonetheless, has the advantage that the probability of failure is an exponential function of exposure as measured by $H(a)$ and, thus, it is more mathematically tractable.}

4b. Environmental Exposure Levels during Different Time Periods

If the environmental exposure level for susceptible *men* during the *1st Time Period* is $\{H(a_1)\}$, and if, as *above*, we define $\{F_m(a) = Zm\}$ as the failure probability for susceptible *men* (i.e., the probability of the event that a randomly selected susceptible *man* develops MS), during some *Time Period* (E_T), and if we define $(\boldsymbol{c})$ as the maximum failure probability for susceptible *men*, then:

$$F_m(a) = Zm = P(MS \mid M, G, E_T) = P(MS, E \mid M, G, E_T)$$

and: $$\boldsymbol{c} = \lim_{a \to \infty}(Zm) = P(MS \mid M, G, E) \leq 1$$

In this case, the failure probability for susceptible *men* during the *1st Time Period* (Zm_1), can be expressed as:

$$F_m(a_1) = Zm_1 = P(MS, E \,|\, M, G)_1 = \boldsymbol{c} * [1 - e^{-H(a_1)}] \qquad (Equation\ S6a)$$

If the exposure level for susceptible *men* during the *2nd Time Period* is $\{H(a_2)\}$, then, because (Zm) is *currently* increasing with time, the difference in exposure for *men* between the *1st and 2nd Time Periods* can be represented as the difference in the environmental exposure level between these two *Time Periods* (q_m) such that:

$$H(a_2) - H(a_1) = q_m > 0$$

In this circumstance, the failure probability for susceptible *men* during the *2nd Time Period* (Zm_2), can be expressed as:

$$F_m(a_2) = Zm_2 = P(MS, E \,|\, M, G)_2 = \boldsymbol{c} * [1 - e^{-\{H(a_1)+q_m\}}] \qquad (Equation\ S6b)$$

Rearrangement of *Equations S6a & S6b* yields:

$$1 - Zm_1/\boldsymbol{c} = e^{-H(a_1)}$$

and: $$1 - Zm_2/\boldsymbol{c} = e^{-\{H(a_1)+q_m\}} \qquad (Equation\ S6c)$$

And dividing the 1st of these two rearranged *Equations* by the 2nd yields:

$$(1 - Zm_1/\boldsymbol{c})/(1 - Zm_2/\boldsymbol{c}) = e^{q_m} \qquad (Equation\ S6d)$$

or: $$q_m = \ln(1 - Zm_1/\boldsymbol{c}) - \ln\,(1 - Zm_2/\boldsymbol{c}) \qquad (Equation\ S6e)$$

Previously, we assigned the value of these arbitrary units as ($q_m = 1$) in these *Equations* [3], although such an assignation may be inappropriate. Thus, this unit (whatever it is) still depends upon the actual (but unknown) level of environmental change that has taken place between the two chosen *Time Periods*. From *Equations S6d–e*, this level depends upon the value of ($\boldsymbol{c}$), which can range over the interval of: ($1 \geq \boldsymbol{c} > Zm_2$) – *see Methods #2D* (*Main Text*). Because (Zm) increases with increasing exposure, the ratio on the *LHS* of *Equation S6d* (*above*) is always greater than unity and it increases monotonically as ($\boldsymbol{c}$) varies throughout its range. This ratio is at its minimum when: ($\boldsymbol{c} = 1$) and approaches infinity as: ($\boldsymbol{c} \rightarrow Zm_2$).

Therefore, we can define (q_m^{min}) as the *"minimum"* possible exposure level change for *men* between these two *Time Periods*. In this case, this *minimum* exposure level change will occur when:

$$\boldsymbol{c} = P(MS \,|\, M, E, G) = 1$$

so that, from *Equation 6e*: $$q_m^{min} = \ln(1 - Zm_1) - \ln\,(1 - Zm_2)$$

However, this *minimum* exposure level change (q_m^{min}) may not accurately characterize the actual (but unknown) level of environmental change, which has taken place for susceptible *men* between the two *Time Periods*. Therefore, we will refer to (q_m) as the *"actual"* exposure-level change, which may be different from this *minimum* exposure-level change such that:

$$q_m \geq q_m^{min}$$

In an analogous manner, we also define $\{F_w(a) = Zw\}$ as the failure probability for susceptible *women* during any *Time Period* and $(\boldsymbol{d})$ as the ultimate failure probability for susceptible *women* such that:

$$F_w(a) = Zw = P(MS \,|\, F, G, E_T) = P(MS, E \,|\, F, G, E_T)$$

and: $$\boldsymbol{d} = \lim_{a \to \infty} (Zw) = P(MS \,|\, M, F, E) \leq 1$$

In this case, because (Zw) is also *currently* increasing with time, the *Equations* for the failure probability in susceptible *women* during the *1st& 2nd Time Periods*, $(Zw_1 \; and \; Zw_2)$, become:

$$F_w(a_1) = Zw_1 = P(MS, E \,|\, F, G)_1 = \boldsymbol{d} * [1 - e^{-K(a_1)}] \qquad (\textit{Equation S7a})$$

and: $$F_w(a_2) = Zw_2 = P(MS, E \,|\, F, G)_2 = \boldsymbol{d} * [1 - e^{-\{K(a_1) + q_w\}}] \qquad (\textit{Equation S7b})$$

where $\{K(a_1)\}$ represents the exposure level in *women* at the *1st Time Period* and (q_w) represents the *"actual"* level of environmental change for *women*, which has taken place between the two *Time Periods* such that:

$$K(a_2) - K(a_1) = q_w > 0$$

In a manner directly analogous to that presented *above* for the development of *Equation S6e*, it is also the case that:

$$q_w = \ln(1 - Zw_1/\boldsymbol{d}) - \ln(1 - Zw_2/\boldsymbol{d}) \qquad (\textit{Equation S7c})$$

Thus, as *above* for susceptible *men*, the *"minimum"* value (q_w^{min}) for the exposure level change in susceptible *women* will occur under those circumstances for which: $(\boldsymbol{d} = 1)$, so that:

$$q_w^{min} = \ln(1 - Zw_1) - \ln(1 - Zw_2)$$

and: $$q_w \geq q_w^{min}$$

4c. Relationships for the Susceptible Population as a Whole

In an analogous manner, we can define the failure, and hazard functions $\{F_p(u) \; and \; b(u)\}$ for the whole susceptible population (*men* and *women*, combined), during any *Time Period*, where:

$$B(a) = \int_0^a b(u) du$$

$$F_p(a) = Zp = P(MS, E \,|\, G) = \boldsymbol{b} * [1 - e^{-\{B(a)\}}]$$

$$\boldsymbol{b} = \lim_{a \to \infty} (Zp) = P(MS \,|\, G, E) \leq 1$$

and*:* $$B(a_2) - B(a_1) = q_p > 0$$

and where: $$P(MS \,|\, G, E) = P(MS, F \,|\, G, E) + P(MS, M \,|\, G, E)$$

$$= P(MS \,|\, F, G, E) * P(F \,|\, G, E) + P(MS \,|\, M, G, E) * P(M \,|\, G, E)$$

Because genotype doesn't depend upon the environment, during any *Time Period*, therefore:

$$(F \mid G, E) = (F \mid G) = p$$

in which case the statement *above* can be re-expressed as:

$$Zp = Zw * (p) + Zm * (1 - p) \qquad (Equation\ S7d)$$

Thus, the failure rates for the entire susceptible population (Zp) can be expressed as a linear combination of the failure rates in *women* (Zw) and *men* (Zm).

4d. Relationship of Failure to True Survival

However, unlike true survival (where everyone dies given sufficient time), the probability of developing MS, either for the subset of susceptible *women* $\{Zw = P(MS, E \mid F, G)\}$ or for the subset of susceptible *men* $\{Zm = P(MS, E \mid M, G)\}$, may not approach 100% as the probability of exposure $\{P(E \mid G, E_T)\}$ approaches unity. Moreover, the limiting value for the probability of developing MS in susceptible *men* $(\boldsymbol{c})$ need not be the same as that in susceptible *women* $(\boldsymbol{d})$. Also, even though the values of the $(\boldsymbol{c})$ and $(\boldsymbol{d})$ parameters are unknown, they are, nonetheless, constants for any disease process, which requires environmental factors as an essential component of disease pathogenesis, and they are independent of whether the hazards are proportional. Finally, the threshold environmental exposure (at which MS becomes possible) must occur at: $P(E \mid G, E_T) = 0$; for one (or both) of these two subsets, provided that this exposure level is possible [3]. If the hazards are proportional, a difference in threshold (λ) can be defined as the difference between the threshold in susceptible *women* (λ_w) and the threshold in susceptible *men* (λ_m) – i.e., $(\lambda = \lambda_w - \lambda_m)$. Thus, if the threshold in *women* is greater than the threshold in *men*, (λ) will be positive and $(\lambda_m = 0)$; if the threshold in *men* is greater than the threshold in *women*, (λ) will be negative and $(\lambda_w = 0)$.

Also, in true survival, both the clock and the risk of death begin immediately at time-zero and continue indefinitely into the future, so that the cumulative probability of death always increases with time. By contrast, here, it may be that the prevailing environmental conditions during some *Time Period* (E_T) are such that: $P(E \mid G, E_T) = 0$; even for quite an extended period (e.g., centuries or millennia). In addition, unlike the cumulative probability of death, here, exposure can vary in any direction with time depending upon the specific environmental conditions during (E_T). Therefore, although the cumulative probability of failure (i.e., developing MS) increases monotonically with increasing exposure, it can increase, decrease, or stay constant with time.

4e. Relationship of the (F:M) Sex Ratio to Exposure

Finally, regardless of (λ), and regardless of any proportionality, during any *Time Period*, the failure probability for susceptible *women* (Zw) can be expressed as:

$$Zw = P(MS, E \,|\, G, F, E_T) = P(E \,|\, G, F, E_T) * \; P(MS \,|\, E, G, F)$$

or: $$Zw = P(E \,|\, G, F, E_T) * \boldsymbol{d}$$

and, similarly, the failure probability for susceptible *men* (Zm) can be expressed as:

$$Zm = P(MS, E \,|\, G, M, E_T) = P(E \,|\, G, M, E_T) * \boldsymbol{c}$$

Dividing the *2nd* of these two *Equations* by the *1st*, during any *Time Period*, yields:

$$Zm/Zw = \{P(E \,|\, G, M, E_T)/P(E \,|\, G, F, E_T)\} * \{\boldsymbol{c}/\boldsymbol{d}\} \qquad (\textit{Equation S8})$$

Consequently, any observed disparity between (Zw) and (Zm), during any *Time Period*, must be due to a difference between *men* and *women* in the likelihood of their experiencing a "*sufficient*" environmental exposure, to a difference between ($\boldsymbol{c}$) and ($\boldsymbol{d}$), or to a difference in both.

Therefore, by assuming that: ($\boldsymbol{c} = \boldsymbol{d} \leq 1$), we are also assuming that any difference in disease expression between susceptible *women* and *men* is due entirely to a difference between susceptible *men* and *women* in the probability of their experiencing a "*sufficient*" environmental exposure, despite the fact that, for every (i), the exposure $\{E_i\}$ is both *population-wide* and fixed during any *Time Period* (E_T). Because this exposure is "*available*" to everyone, therefore, if the level of "*sufficient*" exposure differs between genders, one possibility might be that this is due to a systematic difference in behavior between susceptible *women* and *men* – i.e., to an increased exposure to, or avoidance of, susceptible environments by one or the other gender (perhaps consciously or unconsciously; or perhaps due to differing gender-roles, differing occupations, differing recreational activities, etc.). However, the fact that *most women* behave differently from *men* does not mean that *all women* do so. Notably, if the circumstance of ($\lambda \neq 0$) were explained by a systematic difference in behavior, then the observation of ($\lambda > 0$) suggests that the behavior of *men* leads to a greater exposure than the behavior of *women.* However, any general conclusion regarding such a difference in behavior between susceptible *women* and *men* cannot be rationalized with the observation that, currently: ($Zw_2 > Zm_2$). – *see Section 6g; below*.

Another possible explanation for ($\lambda > 0$), which does not pose this difficulty, is that the distributions of the so-called "critical exposure *intensity*" levels ("thresholds") differ between *men* and *women* (*see Section 6g; below*). In this case, although the same exposure "*intensity*" may be experienced equally by the two genders, this "*intensity*" might be "*sufficient*" for a disproportionate number of *women* or *men*. This possibility is considered subsequently (*see Sections 6g & 8a–b; below*).

Also, regardless of whether the hazards are proportional, and because proportion of *women* among susceptible individuals (p) is a constant (*see Table 2; Main Text*), therefore, for any solution, the ratio (Zw/Zm), during any *Time Period* (E_T), will be proportional to the observed (*F:M*) *sex ratio* during that period (*see Equation S1a*).

4f. Response Curves to Increasing Exposure

Notably, also, because the response curves for both *men* and *women* are exponential, any two points of observation on these curves will define the entire curve (e.g., the values of Zw and Zm during *Time Period #1 and Time Period #2 – see Equations S6a & S6b* and *S7a & S7b*; *above*). Moreover, if these two curves can be plotted on the same *x-axis* (i.e., if *men* and *women* are responding to the same environmental events), the hazards will always be proportional where the values of $(R = q_w/q_m)$ and (λ) are determined from *Equations S11c–d* (*Section 6a, below*).

5. Non-proportional Hazard Models

5a. General Considerations

If the hazard functions for MS in *men* and *women* are not proportional, it is always possible that the "*actual*' exposure level changes for *men* and *women* are each at their *"minimum"* values – i.e., (q_m^{min}) and (q_w^{min}). However, this is a circumstance, which is true if, and only if: $(\boldsymbol{c} = \boldsymbol{d} = 1)$.

Also, in the circumstance of non-proportionality, the various observed and non-observed epidemiological parameter values still limit possible solutions. However, although $(\boldsymbol{c} \leq 1)$ and $(\boldsymbol{d} \leq 1)$ will still be constants, respectively, for *men* and *women*, no information can be learned about them or about their relationship to each other from changes in the (*F:M*) *sex ratio* and $P(MS)$ over time. The observed changes in these parameter values over time could all simply be due to the different environmental circumstances of different times and different places. In this case, also, although *men* and *women* will still each have environmental thresholds, the parameter (λ) – which relates these thresholds to each other – is meaningless, and there is no hazard proportionality factor (R).

Nevertheless, even with non-proportional hazard, the ratio (Zw/Zm), during any *Time Period,* must still be proportional to the observed (*F:M*) *sex ratio* during that *Time Period* (*see Equation S1a; above*) and, if: $\boldsymbol{c} = \boldsymbol{d} \leq 1$, then any observed disparity between (Zw) and (Zm), must be due entirely to a difference between *women* and *men* in the likelihood of their experiencing a "*sufficient*" environmental exposure (E) during that *Time Period* (*see Equation S8; above*).

6. Proportional Hazard Models

6a. General Considerations

By contrast, if the hazards for *women* and *men* are proportional with the proportionality factor (R), the situation is altered. First, because $(R > 0)$, those changes, which take place for the subsets $P(F, MS)$ and $P(M, MS)$ over time, must have the same directionality. Indeed, our epidemiological observations are in accordance with this, where, over the past several decades, the prevalence of MS has been increasing for both *women* and *men* [6,22-30]. Second, including a possible difference in threshold between the genders, the proportionate hazard *Model* can be represented by:

$$\forall\, H(a) > \lambda: \quad K(a) = R * \{H(a) - \lambda\} > 0 \qquad (\textit{Equation S9})$$

In this case, *Equations S7a & S7b*, which represent the failure probability in susceptible *women* given the exposure during the *1st& 2nd Time Periods*, can be re-written as:

$$Zw_1 = \boldsymbol{d} * \left[1 - e^{-K(a_1)}\right] = \boldsymbol{d} * [1 - e^{-R*\{(H(a_1)-\lambda\}}] \qquad (\textit{Equation S10a})$$

and: $$Zw_2 = \boldsymbol{d} * \left[1 - e^{-K(a_2)}\right] = \boldsymbol{d} * [1 - e^{-R*\{H(a_1)+q_m-\lambda\}}] \qquad (\textit{Equation S10b})$$

Rearranging *Equations S6a & S10a* for <u>*any*</u> *Time Period* yields:

$$1 - Zw/\boldsymbol{d} = e^{-K(a)} = e^{-R*\{H(a)-\lambda\}} \qquad (\textit{Equation S11a})$$

and: $$1 - Zm/\boldsymbol{c} = e^{-H(a)} \qquad (\textit{Equation S11b})$$

After dividing *Equation S11a* by *S11b*, we can rearrange this result, to yield:

$$\lambda = \{\ln[1 - Zw/\boldsymbol{d}] - \ln[1 - Zm/\boldsymbol{c}]\}/R + [(R-1)/R] * H(a) \qquad (\textit{Equation S11c})$$

We apply *Equation S11c* to exposure levels $H(a_1)$ and $H(a_2)$ and subtract the 2nd of the resulting two *Equations* from the 1st. Then, using a combination of *Equations S6e & S7c*, together with the definitions of (q_m) and (q_w) from *Section 4b* (*above*), we can rearrange this result to yield:

$$(R-1) * (q_m) = (q_w - q_m)$$

or: $$R = q_w/q_m \qquad (\textit{Equation S11d})$$

Moreover, for those circumstances in which $(R = 1)$, *Equation S11c* becomes:

$$\lambda = \ln[1 - Zw/\boldsymbol{d}] - \ln[1 - Zm/\boldsymbol{c}] \qquad (\textit{Equation S11e})$$

For any specific exposure level $\{H(a) > \lambda\}$, the quantities (Zw) and (Zm) are unknown. However, considering any disease for which a proportionate hazard *Model* is appropriate, the parameters $(\boldsymbol{c}, \boldsymbol{d}, R, \& \lambda)$ are fixed (but unknown) constants, so that, from *Equations S11a & S11b*, the values of (Zm) and (Zw) are also fixed at any specific exposure level $\{H(a)\}$.

6b. Defining an "Apparent" Proportionality Factor

We can also define a so-called "*apparent*" value of the hazard proportionality factor (R^{app}) such that: $R^{app} = (q_w^{min}/(q_m^{min})$, which represents the value (R) when: ($\boldsymbol{c} = \boldsymbol{d} = 1$) – see *Section 5a*; *above*. This value incorporates, potentially, two fundamentally different processes. First, it may capture the increased level of "*sufficient*" exposure experienced by one group compared to the other. Indeed, from *Equation S8*, this is the only interpretation possible for circumstances where: ($\boldsymbol{c} = \boldsymbol{d} \leq 1$). Second, however, if we admit the possibility that: ($\boldsymbol{c} < \boldsymbol{d} \leq 1$), then some of ($R^{app}$) will be accounted for by the difference of ($\boldsymbol{c}$) from unity. For example, when ($\boldsymbol{d} = 1$), and using a proportionate hazard *Model* (*see Section 4b; above & Section 7a; below*), the *"actual"* exposure level change in *men* (q_m) has the limits:

$$q_m^{min} \leq q_m \leq q_w^{min}$$

where: $$\boldsymbol{c} = (Zm_2) * \{e^{q_m} - [P(M, MS)_1/P(M, MS)_2]\}/(e^{q_m} - 1) \leq 1$$

From this, we can define the *"actual"* hazard proportionality factor (R), at ($\boldsymbol{d} = 1$), such that:

$$R^{app} \geq R = q_w^{min} \,/\, q_m$$

In this manner, if ($q_m > q_m^{min}$), a portion of the "*apparent*" value (R^{app}) will be accounted for by a reduction in value of ($\boldsymbol{c}$) from unity, if such a reduction is possible. Moreover, if such a reduction is possible for susceptible *men*, then, clearly, it is also possible that the value of ($\boldsymbol{d}$) is also reduced from unity in susceptible *women*. For example, in circumstances where: ($\boldsymbol{c} < \boldsymbol{d} < 1$), the *"actual"* exposure level in *women* (q_w) would be greater than its minimum value (q_w^{min}) such that:

$$R = q_w/q_m > q_w^{min}/q_m$$

Consequently, in each of these circumstances, the *"actual"* value of (R) may be different from its *"apparent"* value (R^{app}).

{NB: Considering Equations S6a & S10a for any Time Period (above), the ($\boldsymbol{c}$) and ($\boldsymbol{d}$) constants, although defined differently (see Section 4b; above), are each the equivalent of a y-axis "scaling factor" for their respective exponential curves, considered separately. However, when ($\boldsymbol{c} = \boldsymbol{d}$), this "scaling factor" is the same for both men and women and, therefore, under those conditions where: ($\boldsymbol{c} = \boldsymbol{d} \leq 1$), all response curves for both men and women, which have the same (R) and (λ), will differ from each other only in that the y-axis scale is different. For example, suppose that we define a constant (α) such that: $\boldsymbol{c} = \boldsymbol{d} = \alpha < 1$. In this case, the response curves for men and women, depicted after changing the y-scale by multiplying both sides of Equations S6a & S10a (for any Time Period) by the constant ($1/\alpha$), are identical to those curves having the same (R) and (λ), but depicted for: ($\boldsymbol{c} = \boldsymbol{d} = 1$).

This does not imply that these response curves are "actually" identical. Rather, the fact that the curves for those conditions under which: ($\boldsymbol{c} = \boldsymbol{d} = \alpha < 1$) can be scaled to be identical to those depicted for:

$(\boldsymbol{c} = \boldsymbol{d} = 1)$, *only indicates that the relationship* <u>*between*</u> *the response curve for men and that of women is the same regardless of the value of* (α) *– i.e., any changes in the* (Zw/Zm) *ratio with increasing exposure or, equivalently, any changes of the F:M sex ratio (see Equations S1a & S8; above), will be the same for any value of* (α). *When* $(\boldsymbol{c} < \boldsymbol{d})$, *this kind of transformation is not possible.}*

6c. Implications that the $(\boldsymbol{R})$ Value has for the Values of $(\boldsymbol{\lambda})$, $(\boldsymbol{c})$ and $(\boldsymbol{d})$

Assertions: *1.* $\forall(R \geq 1)$: $\lambda > 0$

2. $\forall(R \leq 1)$: $\boldsymbol{c} < \boldsymbol{d} \leq 1$

Proof: We define the ratios $(C_F$ & $C_M)$ – *see Table 2; Main Text* – and note that, because both $P(MS)$ and the (*F:M*) *sex ratio* are both increasing with time [6,22-30], therefore:

$$C_F = P(F, MS)_1/P(F, MS)_2 < P(M, MS)_1/P(M, MS)_2 = C_M$$

Notably, also, from *Equation S1a*, during any *Time Period*:

$$(F{:}M)\ sex\ ratio = (Zw/Zm) * \{p/(1-p)\}$$

where (p) is independent of the environmental conditions of any *Time Period*. Therefore, for all solutions, the ratio (Zw/Zm) will mirror (*F:M*) *sex ratio* (i.e., the changes in both ratios will have the same directionality).

1. <u>*For those Conditions in which:* $(R = 1)$:</u>

From *Section 6a* (*above*) for circumstances where: $\{R = (q_w/q_m) = 1\}$, it must be that:

$$q_m = q_w \geq q_w^{min}$$

When: $(\lambda = 0)$, from *Equation S11e* (*above*):

$$Zm/\boldsymbol{c} = Zw/\boldsymbol{d}$$

or: $$Zw/Zm = \boldsymbol{d}/\boldsymbol{c} \qquad (\textit{Equation S12a})$$

Therefore, in this circumstance, the *F:M sex ratio* will remain constant, regardless of the exposure level.

However, from the *Summary Equations* presented in *Section 7a* (*below*):

$$\boldsymbol{d}/\boldsymbol{c} = \{Zw/Zm\} * \{(e^{q_w} - C_F)/(e^{q_w} - C_M)\} > Zw/Zm$$

This relationship indicates that: $Zm/\boldsymbol{c} > Zw/\boldsymbol{d}$

So that, from *Equation S11e*: $\lambda > 0$

Thus, if $(R = 1)$, and if both the *F:M sex ratio* and $P(MS)$ are increasing, it must be the case that the threshold in susceptible *women* is greater than that in susceptible *men.*

2. <u>*For those Conditions in which:* $(\lambda \leq 0)$ & $(R > 1)$:</u>

For $\{H(a) > 0\}$, thus, from *Equation S11c*, under these conditions, for any *Time Period*:

$$\{\ln(1 - Zw/\boldsymbol{d}) - \ln(1 - Zm/\boldsymbol{c})\}/R = \lambda - [(R-1)/R] * H(a) < 0$$

or: $$\ln(1 - Zw/\boldsymbol{d}) - \ln(1 - Zm/\boldsymbol{c}) < 0 \qquad (\textit{Equation S12b})$$

In turn, *Equation S12b,* in this circumstance, requires that:

$$Zm/\boldsymbol{c} < Zw/\boldsymbol{d}$$

or: $$Zw/Zm > \boldsymbol{d}/\boldsymbol{c} \qquad (\textit{Equation S12c})$$

Moreover, regardless of the value of (R), from the definitions of (E), $(\boldsymbol{c})$, and $(\boldsymbol{d})$, and from *Equation S8* (*above*):

$$\lim_{a\to\infty}(Zw/Zm) = \boldsymbol{d}/\boldsymbol{c} \qquad (\textit{Equation S12d})$$

Therefore, because both (Zw) and (Zm) increase monotonically with increasing exposure (*see Section 4a; above*), and because $(R > 1)$, and because $(\lambda \leq 0)$, and because $\{H(a) > 0\}$, the condition that:

$$Zw/Zm > \boldsymbol{d}/\boldsymbol{c}$$

requires that: $Zw_1/Zm_1 > Zw_2/Zm_2 > \boldsymbol{d}/\boldsymbol{c}$:

so that, in this circumstance, the ratio (Zw/Zm) decreases with increasing exposure. Because the (Zw/Zm) ratio mirrors the *F:M sex ratio* (*see above*), therefore, under these conditions, the *F:M sex ratio* will also be decreasing (e.g., *Figures 1A–B; Main Text*) – a conclusion, which is contrary to the available evidence [6,22-30]. Consequently, the conditions: $(R > 1)$ & $(\lambda \leq 0)$ are not plausible, given the Canadian data [6].

Combining the conclusions from *Conditions 1 & 2* (*above*), therefore, it is clear that:

$$\forall(R \geq 1):\ \lambda > 0$$

Thus, based solely on the observation that both $P(MS)$ and the (*F:M*) *sex ratio* are increasing with time [6,22-30] – a circumstance which is true considering the "*current*" *Time Period #2* together with any of the reported previous 5-year epochs as *Time Period #1* [6] – we can conclude, based on purely theoretical grounds that, if the hazards are proportional, and if: $(R \geq 1)$, susceptible *women* must have a higher threshold than susceptible *men.*

3. <u>*For those Conditions, in which*</u>*:* $(\lambda \geq 0)$ & $(R \leq 1)$

If: $(\lambda \geq 0)$ & $(R \leq 1)$ & $(\boldsymbol{c} = \boldsymbol{d} \leq 1)$; then *men* would have as great (or a greater) failure probability than *women* (i.e., $Zm \geq Zw$) at every exposure level (e.g., *Figure 1C; Main Text*). However, because: $(Zw_2 > Zm_2)$ – see *Section 2c* (*above*); *see also Figure 2B; Main Text* – these conditions are not possible. Therefore, whenever: $(\lambda \geq 0)$ & $(R \leq 1)$, then: $(\boldsymbol{c} < \boldsymbol{d} \leq 1)$ – e.g., *Figure 1D* (*Main Text*).

4. <u>*For those Conditions, in which*</u>*:* $(\lambda < 0)$ & $(R \leq 1)$:

In this circumstance, *Equation S12b* still holds so that; if: $(\boldsymbol{c} = \boldsymbol{d} \leq 1)$, then: $(Zm > Zw)$ at every exposure level after the response curve in men intersects that in women (e.g., *Figures 2A&B; Main Text*). Therefore, again, because the conditions of: $(Zw_2 > Zm_2)$ and an increasing *F:M sex ratio* only occur together after the intersection, the posited conditions are not possible. However, it is still possible that: $(\boldsymbol{c} < \boldsymbol{d} \leq 1)$ – e.g., *Figure 2D* (*Main Text*).

5. *For those Conditions, in which:* $(R^{app} > 1)$:

The proportionality constant (R) relates to how quickly the response curves for *men* and *women* go from zero to their maximums. As such, the value of (R) is independent of (λ) but, rather, depends only upon how quickly this transition occurs. Therefore, we are free to choose, for comparison, the response curves at any value of (λ). In this case, when $(\boldsymbol{c} = \boldsymbol{d})$ & $(\lambda = 0)$, *Equations S6a & S10a*, for any *Time Period*, can be multiplied by the scaling factor of: $(1/\boldsymbol{c})$, and then restated such that:

$$Zm/\boldsymbol{c} = (1 - e^{\{H(a)\}})$$

and: $$Zw/\boldsymbol{c} = (1 - e^{R*\{H(a)\}})$$

Clearly, the *RHS* of both *Equations* is independent of scale. Moreover, the relationship of the *LHS between* the two *Equations* is also independent of scale. Consequently, the relationship *between* each *Equation*, when $(\boldsymbol{c} = \boldsymbol{d})$, is independent of scale (*see Note; Section 6b; above*). Therefore, in the circumstance where: $(\boldsymbol{c} = \boldsymbol{d})$ the value of (R) is constant for all: $(Zm_2 < \boldsymbol{c} \leq 1)$. Consequently:

$$\forall(\boldsymbol{c} = \boldsymbol{d}):\ R^{app} = q_w^{min}/q_m^{min} = q_w/q_m = R$$

However, if conditions are such that: $(R \leq 1)$, then also, $(q_w \leq q_m)$.

Therefore, whenever: $(R^{app} > 1)$, then:

$$R^{app} = q_w^{min}/q_m^{min} > 1 \geq q_w/q_m = R$$

Thus, the observation that: $(R^{app} > 1)$, also implies that:

$$\forall(R \leq 1):\ \boldsymbol{c} < \boldsymbol{d}$$

Combining the conclusions *Conditions 3–5* (*above*), it is clear that:

$$\forall(R \leq 1):\ \boldsymbol{c} < \boldsymbol{d} \leq 1$$

In fact, following a similar logic to that presented *above*, it must be the case that:

$$\forall(R^{app} > R):\ \boldsymbol{c} < \boldsymbol{d}$$

Also, combining the conclusions from *Conditions 1–5* (*above*), it is clear that, whenever: $(\boldsymbol{c} = \boldsymbol{d} \leq 1)$, then, on theoretical grounds, it must also be the case that both: $(R > 1)$ and: $(\lambda > 0)$.

6d. Strictly Proportional Hazard: $(\boldsymbol{\lambda} = \boldsymbol{0})$

If the hazards in *men* and *women* are "strictly" proportional to each other, then it must be the case that: $(\lambda = 0)$. Therefore, when: $(|\lambda| > 0)$, as it must be when $(R \geq 1)$, the hazards cannot be "strictly" proportional. Indeed, for those circumstances in which $(R \geq 1)$ and $(\lambda = 0)$, the observed (*F:M*) *sex ratio*, as discussed *above*, either decreases or remains constant with increasing exposure (*see Equations S12a–c; above*), regardless of the parameter values for $(\boldsymbol{c})$ and $(\boldsymbol{d})$ – e.g., *Figures 1A & 1B* (*Main Text*). Consequently, the only "strictly" proportional circumstances, which are possible, are those in which *men* have a greater hazard than *women* – i.e., $(R < 1)$. Moreover, if *men* have a greater hazard than *women*, then, as noted *above*, the conditions of: $(\boldsymbol{c} = \boldsymbol{d} \leq 1)$ & $(\lambda = 0)$ are also excluded.

Thus, the only possible "strictly" proportional circumstances are those, in which both $(R < 1)$ and $(\boldsymbol{c} < \boldsymbol{d} \leq 1)$ – e.g., *Figure 1D* (*Main Text*).

{NB: In these and subsequent Figures, all response curves exemplifying the conditions in which $(\boldsymbol{c} = \boldsymbol{d} \leq 1)$*, are depicted for the condition* $(\boldsymbol{c} = \boldsymbol{d} = 1)$*. Nevertheless, for all those conditions where* $(\boldsymbol{c} = \boldsymbol{d} < 1)$*, the response curves differ from the curves depicted in the Figures only in so far as the y-axis has a different scale. Therefore, the response curves, depicted at:* $(\boldsymbol{c} = \boldsymbol{d} = 1)$*, are representative of all curves for which* $(\boldsymbol{c} = \boldsymbol{d})$ *– see Section 6c (above).}*

6e. Intermediate Proportional Hazard: $(\boldsymbol{\lambda} < \mathbf{0})$

We can also consider another possible *Model*, which is intermediate between the "strictly" proportional and non-proportional hazard *Models* discussed *above*. In this intermediate *Model*, the hazards are still held to be proportional although the onset of the response curves are offset from each other by an amount $(\lambda \neq 0)$. As noted earlier: $\forall\ (R \geq 1): \lambda > 0$. Therefore, for those circumstances in which $(\lambda < 0)$, the hazard in *men* must be greater than the hazard in *women*. Moreover, under those conditions, for which $(\boldsymbol{c} = \boldsymbol{d} \leq 1)$ & $(R < 1)$ & $(\lambda < 0)$, the (*F:M*) *sex ratio* will decrease with increasing exposure until the two response curves have intersected (e.g., *Figures 2A & B; Main Text*), reaching a level below $\{p/(1-p)\}$. Following this, the (*F:M*) *sex ratio* steadily increases to ultimately reach the level of $\{p/(1-p)\}$. However, after the response curve in *men* has intersected that in *women* (i.e., after this nadir), this circumstance requires that $(Zm > Zw)$ throughout the entire remaining response curve until an (*F:M*) *sex ratio* of: $\{p/(1-p)\}$ is reached (e.g., *Figures 2A & B; Main Text*). The only circumstance in which the *Model* of $(\boldsymbol{c} = \boldsymbol{d} \leq 1)$ would work is one in which (p) is at least as large as the "*current*" value of (p') – i.e., $(p'_2 \geq 0.74)$; *see Section 2c* (*above*). Each of these possibilities is contrary to evidence where *currently* $(Zw_2 > Zm_2)$ and, thus, where: $\{(F{:}M)\ sex\ ratio > p/(1-p)\}$ – *see Equation S1a* (*above*). Thus, the condition of: $(\lambda < 0)$ is only possible, in circumstances where: $(\boldsymbol{c} < \boldsymbol{d})$ – e.g., *Figure 2D* (*Main Text*).

6f. Intermediate Proportional Hazard: $(\boldsymbol{\lambda} > \mathbf{0})$

By contrast, when $(\lambda > 0)$, although the hazard is still proportional, there are no absolute constraints on the hazard in *men* relative to that in *women*. Thus, the conditions of: $(R < 1)$ & $(\lambda \geq 0)$ – e.g., *Figures 1D & 4C* (*Main Text*) – and: $(R \geq 1)$ & $(\lambda > 0)$ – e.g., *Figures 3 & 4* (*Main Text*) – can each lead to very similar conclusions. For this discussion, we define (G_{ia}) to be the susceptibility genotype of the ith susceptible individual that includes all (and only) those genetic factors, related to MS susceptibility, which are located on autosomal chromosomes. Also, the occurrence of (G_{ia}) represents the event that an autosomal genotype, randomly selected from all such genotypes within (Z), is a member of the (G_{ia}) subset. We define the family $\{G_a\}$ to include all the subsets (G_{ia}) within the (G) subset. In a similar manner, the occurrence of $\{G_a\}$

represents the event that an autosomal genotype, randomly selected from all such genotypes within (Z), is a member of the $\{G_a\}$ family. The "susceptibility" genotype (G_{is}), defined previously (*Methods #1A, Main Text*), includes all (and only) those genetic factors, which are related to MS susceptibility (located on any chromosome) but does not include the entire genotype of the *i^th^* individual. The event (G_{is}) and the family $\{G_s\}$ have also been defined previously (*see Methods #1A; Main Text*). Because (G_{ia}) genotypes are purely autosomal, we expect that:

$$P(G_{ia} \,|\, M) = P(G_{ia} \,|\, F)$$

$$P(G_{ia}, F, G_{is}) = P(F, G_{is})$$

and: $$P(G_{ia}, M, G_{is}) = P(M, G_{is})$$

Naturally, *women* and *men* may be members of the same (G_{ia}) subset, but not be members of the same (G_{is}) subset, either if there are factors on the *X*-chromosome related to susceptibility that differ between some susceptible *men* and *women*, or if there are factors on the *Y*-chromosome in some *men*, which are related to susceptibility. Therefore, these equivalences do not necessarily imply either that:

$$P(F, G_{is}) = P(M, G_{is})$$

or that both: $P(F, G_{is}) > 0$ and: $P(M, G_{is}) > 0$

Nevertheless, of the 233 genetic loci, which have been reproducibly identified by the *International Multiple Sclerosis Genetics Consortium* as being MS-associated, all but one are located on autosomal chromosomes; and even for the single locus found on the X-chromosome, *men* and *women* were both found to carry the risk-variant [60]. In this circumstance, therefore, it seems likely that:

$$(F, G_{ia}) \approx (F, G_{is}) \quad \text{and:} \quad (M, G_{ia}) \approx (M, G_{is})$$

and, consequently, the same will be true for "*i-type*" groups such that:

$$(F, G_{ia}) \approx (F, G_{it}) \quad \text{and:} \quad (M, G_{ia}) \approx (M, G_{it})$$

and therefore, in most circumstances, both *men* and *women* can (at least potentially) belong to the same "*i-type*" group. This conclusion is also supported the available genetic evidence (*see Discussion Section; Main Text*). Moreover, in this conceptualization, the environmental factors that comprise each set of "*sufficient*" exposures within the $\{E_i\}$ family (for *i-type* individuals) are envisioned to be the same regardless of whether the "*i-type*" individual happens to be a *man* or a *woman* – except that, when: $(\lambda > 0)$, a "*sufficient*" exposure for an "*i-type*" *woman* may need to be more "*intense*" than it is for an "*i-type*" *man* (*see Section 6g; below*).

6g. Considerations of Exposure "Intensity"

In considering the notion of exposure "*intensity*", three conclusions seem to be well established. First, for every proportional hazard solution that we identified (*see Results Section; Main Text*), we found that: $(R^{app} > 1)$. Moreover, as demonstrated on theoretical grounds in *Sections 6b–c* (*above*), and as depicted in *Figure S1* (*below*) *& Figure 4; Main Text*, in these circumstances, it must be that:

$$\forall (R \leq 1): \; \boldsymbol{c} < \boldsymbol{d}$$

Second, as demonstrated in *Section 6c* (*above*), under those circumstances, in which both $P(MS)$ and $P(F \mid MS)$ are increasing with time, then:

$$\forall(R \geq 1):\ \lambda > 0$$

Third, from the Canadian data [6], it seems inescapable that, as the probability of a "*sufficient*" exposure for susceptible individuals has increased over the past several decades, the probability of developing MS for susceptible *women* has increased at a faster rate than it has for susceptible *men*. Consequently, if the hazards in *men* and *women* are proportional, this faster rate of increase in susceptible *women* implies that one of the following two conditions must hold. Thus, either:

1) $R \leq 1$ in which case: $\boldsymbol{c} < \boldsymbol{d}$

or: 2) $R > 1$ in which case: $\lambda > 0$

Clearly, the first of these conditions excludes the possibility that: $\boldsymbol{c} = \boldsymbol{d} = 1$

In considering the second of these conditions, it should be noted that both of our measures of exposure – i.e., (a) and $H(a)$ – relate directly back to the parameter $P(E \mid G)$, which represents the probability of the event that a randomly selected susceptible individual (either a *man* or a *women*) experiences an environmental exposure "*sufficient*" to cause MS in them. Therefore, this second condition – i.e., that: $\lambda > 0$ – indicates that, as the probability of a "*sufficient*" exposure decreases, there comes a point where only susceptible *men* can develop MS. This implies that, at (or below) this point: $(R \approx 0)$. Consequently, the requirement that $(R > 1)$ creates a paradox in that, for the second condition to be true, susceptible *women* must be more likely than *men* to experience a "*sufficient*" exposure when the probability $\{P(E \mid G)\}$ is high and, yet, susceptible *men* must be much more likely than *women* to experience a "*sufficient*" exposure when this probability is low.

There are two obvious ways to avoid this paradox. The first is to conclude that the hazards are not proportional. Nevertheless, despite this possibility, such a conclusion also presents problems of its own (*see Discussion Section; Main Text*). For example, because *women* and *men* of the same "*i-type*" necessarily have proportional hazards (*see Section 6h; below*), in this case, we would also have to conclude that susceptible *women* and *men* can never be in the same "*i-type*" group and, therefore, that each gender requires distinct sets of environmental conditions to develop MS. Thus, we would have to further conclude that MS in *women* must represent a disease distinct from MS in *men*. Alternatively, if susceptible *women* and *men* could both be members of certain "*i-type*" groups but not others, we would have to conclude MS represents three distinct diseases (one in *women*, one in *men*, and a third in both). Any such conclusion seems to be at substantial variance with both the genetic and the epidemiological evidence (*see Discussion Section; Main Text*).

The second way to avoid the paradox, is to conclude that the first of the two possible proportional hazard conditions is true – i.e., that both $(R \leq 1)$ and: $(\boldsymbol{c} < \boldsymbol{d})$. Notably, the condition of: $(R \leq 1)$ is compatible with any value of (λ). However, if $(\lambda > 0)$, the simultaneous condition of: $(R \leq 1)$, offers, at least, a more consistent interpretation of the existing data. Thus, under these conditions, at every population

exposure level (a), the probability of the event that a randomly selected susceptible *man* will experience a "*sufficient*" environmental exposure to cause MS in them is as great, or greater, than the same probability for a susceptible *woman* (*see Figure S1 & Figures 4 & 5; Main Text*). Thus, although the notion of a "critical exposure *intensity*" (*discussed below*) may be necessary to rationalize any threshold difference, it is not necessary to resolve a paradox. Nevertheless, accepting this conclusion, does require also accepting the fact that some susceptible *men* will never develop MS, even when the correct genetic background occurs together with an environmental exposure "*sufficient*" to cause MS in them.

Different Meanings of Exposure "Intensity"

When considering any other circumstance, for which $(\lambda > 0)$, it is important to distinguish between the "*intensity*" (or level) of exposure as measured by the odds (a) – *see Section 4a; above* – and the "*intensity*" (or level) of exposure to the individual factors or events that, together, comprise the "*sufficient*" sets within each $\{E_i\}$ family. Thus, as noted above, both of our measures of exposure – i.e., (a) and $H(a)$ – relate directly to the probability $\{P(E \mid G)\}$. We describe this increasing probability as an increasing "*intensity*" of exposure. Also, because each "*i-type*" group is different, we define (λ_i) to be the difference in threshold between "*i-type*" *women* and "*i-type*" *men.*

Exposure "Intensity" for a Single Factor

In this context, it is helpful, initially, to consider an example using a markedly over-simplified pathogenetic *Model* for MS. In this simplified *Model*, a single factor (e.g., vitamin D deficiency) is held to be the sole environmental factor responsible, by itself, for causing MS in both susceptible *women* and *men.* In this case, we can imagine a circumstance (e.g., *Figure 5A; Main Text*), in which the population vitamin D levels are such that no susceptible person (or "*i-type*") has any chance of having a "*sufficient*" deficiency – i.e., where: $\{P(E \mid G) = 0\}$. As the population vitamin D levels drop and deficiency becomes more prevalent in the population, it finally reaches the point where the levels can be low enough (e.g., *Figure 5B; Main Text*) such that some "*i-types*" begin to have some chance of developing MS – i.e., where: $\{0 < P(E \mid G) < 1\}$. We can define the vitamin D level, at which the deficiency becomes "*sufficient*" to cause MS in a particular "*i-type*" both as the "critical exposure *intensity*", or the "threshold", level for that "*i-type*". We can also define this level to be the "critical" exposure level for the single factor (e.g., vitamin D deficiency) for that "*i-type*". For this simple pathogenic *Model*, these two definitions are the identical whereas, for *Models* with more factors, these may be different (*see below*). As vitamin D levels in the population decline further (i.e., as the exposure become more "*intense*"), more and more "*i-types*" will reach their "critical exposure *intensity*" level (e.g., *Figure 5C; Main Text*) until, finally (e.g., *Figure 5D; Main Text*), the population vitamin D deficiency becomes severe enough such that everyone experiences a "*sufficient*" exposure – i.e., where: $\{P(E \mid G) = 1\}$.

In this circumstance, any "*i-type*" with a lower "critical exposure *intensity*" level compared to some

other “*i-type*”, will also have a greater probability of experiencing a “*sufficient*” vitamin D deficiency at every level of population exposure less than the *maximum – see Sections 8a–b; Figures S1–S3*. Consequently, in this simple *Model*, the population exposure “*intensity*” (as measured by vitamin D levels) is directly related to the “*intensity*” as measured by each of our metrics – i.e., $P(E \mid G)$, (a) or $H(a)$. Moreover, in this case, because *women* and *men* are responding to the same environmental event, the value of (λ) is simply a reflection of the difference in “critical exposure *intensity*” level between those susceptible “*i-type*” *women* (who require the least deficiency of any *woman*) and those susceptible “*i-type*” *men* (who require the least deficiency of any *man*). However, the value of the proportionality constant (R) cannot be predicted from the “*intensity*” of exposure (a) because this constant is determined by measuring exposure as the integral of an unknown hazard function rather than as (a) – *see note in Section 4a* (*above*). Nevertheless, the probability distributions for the *log-transformed* “critical” (or threshold) levels of exposure for susceptible individuals (*women* and *men* considered separately), will have, respectively, both means (μ_w and μ_m) and variances (σ_w^2 and σ_m^2) for each distribution. In this case, the variance of each distribution determines how rapidly probability of a “*sufficient*” exposure reaches any point on the *y-axis* (*see Figures S1–S3*), which is also exactly what the parameter (R) determines. Therefore, while, in general, it is true the value of (R) cannot be determined exactly in these circumstances, nevertheless, when: $(\sigma_w^2 = \sigma_m^2)$; then: $(R = 1)$ – *e.g., Figure S1; Section 8b & Figure 5; Main Text*; when $(\sigma_w^2 > \sigma_m^2)$, then: $(R < 1)$ – *see legend of Figure S1; Section 8b*; and, when: $(\sigma_w^2 < \sigma_m^2)$; then: $(R > 1)$ – *e.g., Figure S2 & S3* (*Section 8b*).

Exposure “Intensity” for Multiple Factors

However, in contrast to this simplified *Model*, MS pathogenesis is known to involve multiple environmental factors [3,9], in which case, the relationship between our exposure metrics – $P(E \mid G)$, (a) or $H(a)$ – and the “*intensity*” level for any individual factor (however these are determined) becomes less clear. As discussed above, here, we are measuring exposure as the probability of the event that a randomly selected susceptible individual experiences an environment “*sufficient*” to cause MS in them given the environmental cri conditions of the time (E_T). This measure does not depend upon any specific environmental conditions. Rather it indicates only that those environmental conditions, which exist at some point in time, result in this probability. Nevertheless, regardless of these uncertainties, if each factor can vary in their “critical” exposure level from one “*i-type*” group to another, there must be <u>some</u> relationship between this probability and the individual “critical” exposure level for each factor. Thus, if each factor level is at its <u>*minimum*</u> “*intensity*”, then, in this case, presumably: $\{P(E \mid G) = 0\}$. Conversely, if each factor level is at its <u>*maximum*</u> “*intensity*”, then presumably: $\{P(E \mid G) = 1\}$.

Without knowing the actual environmental factors involved in MS pathogenesis and how they interact, it is difficult to know how this probability might relate to the “*intensity*” of exposure to the different factors. Nevertheless, some possible relationships between this probability and factor “*intensity*” levels can be

envisioned. For example, suppose that the family $\{E_i\}$ includes only one "*sufficient*" set, consisting of (b) environmental factors or events, each of which has some "critical" exposure level – i.e., the exposure level, at (or above) which, the exposure becomes "*sufficient*" for this factor, in this set of exposures, for this "*i-type*" group. The terms: $\{EF_{1i}, EF_{2i}, \ldots and\ EF_{bi}\}$, represent the "critical" exposure levels for each environmental factor $\{EF\}$. By contrast, the round-bracketed terms $(EF_{1i}), (EF_{2i}), \ldots and\ (EF_{bi})$ represent the events that the exposure for a randomly selected "*i-type*" individual has reached (or exceeded) the "critical" exposure level for a particular environmental factor and $P(EF_{1i}), P(EF_{2i}), \ldots and\ P(EF_{bi})$ represent the probabilities of these events. We also assume that *every* "*i-type*" group requires the *same* set of environmental events but that each differs with respect to their "critical" exposure levels for each factor. Moreover, we assume that whether an "*i-type*" individual experiences a "*sufficient*" exposure to one factor or event, is independent of whether or not they experience a "*sufficient*" exposure to any other factor or event. In this case, (E_i) becomes the event that a randomly selected "*i-type*" individual, experiences a "*sufficient*" exposure to every necessary factor such that:

$$(E_i) = (EF_{1i}) \cup (EF_{2i}) \cup \ldots \cup (EF_{bi})$$

so that the probability of (E_i) occurring during the *Time Period* (E_T) is:

$$P\left(E_i \,\middle|\, E_T\right) = P\left(EF_{1i} \,\middle|\, E_T\right) * P\left(EF_{2i} \,\middle|\, E_T\right) * \ldots * P\left(EF_{bi} \,\middle|\, E_T\right) \qquad \textit{Equation S12e}$$

To explain $(\lambda > 0)$, we suppose that some susceptible "*i-type*" *women*, compared to "*i-type*" *men*, have a higher "critical" exposure level to one or more of the factors in this set (i.e., if these factors are "gender-dependent"). Thus, if these "gender-dependent" factors are not already above their *maximum* "*intensity*" levels for a given "*i-type*", $P(E_i)$ will always be smaller in *women* compared to *men*. Moreover, if these differences in $P(E_i)$ between *men* and *women* varied between "*i-types*", one can imagine that the distribution the $P(E_i)$ levels for *men* and *women* might differ either in their means, in their variances, or in both. To explain a consistent $(\lambda > 0)$, however, it is necessary for *men* in some "*i-type*" groups, during any *Time Period* (E_T), to have a smaller "critical exposure *intensity*" level compared to any susceptible *woman* in the population and, thus, for all these "*i-type*" groups to have $(\lambda_i > 0)$. Nevertheless, other "*i-type*" groups could vary in the relative likelihood of experiencing their "critical exposure *intensity*" levels, depending upon the environmental conditions that prevail during (E_T).

Another possibility is to assume the same circumstances as those described *above* except that, in this case, each "*i-type*" group is posited to consist of only a single individual (or two in the special case of *MZ*-twins). Clearly, with only one person (or an *MZ*-twin pair) per "*i-type*" group, the concept of (λ_i) is meaningless. Moreover, in this circumstance, we can define a *minimum* "critical" exposure level for each factor, considering every "*i-type*", such that:

$$EF_{1m} = min\{EF_{1i}\},\ EF_{2m} = min\{EF_{2i}\}, \ldots,\ EF_{bm} = min\{EF_{bi}\}$$

and we can also define a so-called "probability of *minimal* exposure", $\{P(E_m)\}$, such that:

$$P\left(E_m \,\middle|\, E_T\right) = P\left(EF_{1m} \,\middle|\, E_T\right) * P\left(EF_{2m} \,\middle|\, E_T\right) * \ldots * P\left(EF_{bm} \,\middle|\, E_T\right)$$

Thus, $\{P(E_m \,|\, E_T)\}$, is both a constant and, also, the *minimum* possible "critical exposure *intensity*" during any *Time Period* (E_T). Therefore, it must be the case that:

$$\forall(i): \ P(E_m \,|\, E_T) \leq P(E_i \,|\, E_T)$$

We then define constants (a_{ji}) and (b_{ji}) such that:

$$\forall(j = 1,2,\dots,b) \ \& \ \forall(i = 1,2,\dots,m):$$

$$P(EF_{ji}, G_i \,|\, F, E_T) = a_{ji} * \ P(E_{jm} \,|\, E_T)$$

$$P(EF_{ji}, G_i \,|\, M, E_T) = b_{ji} * \ P(E_{jm} \,|\, E_T)$$

where: $\forall G_i \in (F, G)$: $a_{ji} \geq 1$ & $b_{ji} = 0$

and: $\forall G_i \in (M, G)$: $a_{ji} = 0$ & $b_{ji} \geq 1$

so that: $\forall(i = 1,2,\dots,m)$; and: $\forall(a_{ji} \neq 0)$:

$$P(E_i, G_i \,|\, F, E_T) = (a_{1i} * a_{2i} * \dots * a_{bi}) * \ P(E_m \,|\, E_T)$$

Therefore, for the (mp) susceptible *women* in the population (Z):

$$\begin{aligned} P(E \,|\, F, E_T) &= E(E_i, G_i \,|\, F, E_T) \\ &= \left(\tfrac{1}{mp}\right) * \ P(E_m \,|\, E_T) * \textstyle\sum_{i=1}^{m} (a_{1i} * a_{2i} * \dots * a_{bi}) \\ &= P(E_m \,|\, E_T) * E(a_{1i} * a_{2i} * \dots * a_{bi}) \end{aligned}$$

and, also: $\forall(i = 1,2,\dots,m)$; and: $\forall(b_{ji} \neq 0)$:

$$P(E_i, G_i \,|\, M, E_T) = (b_{1i} * b_{2i} * \dots * b_{bi}) * \ P(E_m \,|\, E_T)$$

Similarly, for the $\{m(1 - p)\}$ susceptible *men* in the population (Z):

$$\begin{aligned} P(E \,|\, M, E_T) &= E(E_i, G_i \,|\, M, E_T) \\ &= \left(\tfrac{1}{m(1-p)}\right) * \ P(E_m \,|\, E_T) * \textstyle\sum_{i=1}^{m} (b_{1i} * b_{2i} * \dots * b_{bi}) \\ &= P(E_m \,|\, E_T) * E(b_{1i} * b_{2i} * \dots * b_{bi}) \end{aligned}$$

Because: $\{E(a_{1i} * a_{2i} * \dots * a_{bi})\}$ and: $\{E(b_{1i} * b_{2i} * \dots * b_{bi})\}$ are both fixed population parameters, therefore, during any *Time Period* (E_T), the probability of a "*sufficient*" exposure for susceptible *women* and *men* are proportional such that:

$$P(E \,|\, F, E_T)/P(E \,|\, M, E_T) = E(a_{1i} * a_{2i} * \dots * a_{bi})/E(b_{1i} * b_{2i} * \dots * b_{bi})$$

Again, because (R) is determined from the exposure measure of $\{H(a)\}$, its value cannot be estimated in these circumstances.

Also, in this case, to explain a condition where: $(\lambda > 0)$, then, for some number (M_s) of susceptible *men* $(k = 1,2, \ldots M_s)$, it must be the case that:

$$\forall(i) \;\&\; \forall(k) \;\&\; \forall(E_T):\; 1 \leq (b_{1k} * b_{2k} * \ldots * b_{bk}) < (a_{1i} * a_{2i} * \ldots * a_{bi})$$

It is possibilities such as these, which are depicted in *Figure 5* (*Main Text*) & *Figures S1-S3* (*Sections 8a–b*).

Alternatively: 1) if certain factors are important determinants for some "*i-type*" groups but not for others, or: 2) if different sets within an $\{E_i\}$ family involved different environmental factors, then it is difficult to rationalize any consistent value for $(\lambda \neq 0)$ based on our "*intensity*" measure $\{H(a)\}$. However, regardless of any explanation, if this notion of a "critical exposure *intensity*" level is appropriate and if $(\lambda > 0)$, it must be that some susceptible *men*, during any *Time Period* (E_T), must have a lower "critical exposure *intensity*" level compared to every susceptible *women*. Notably, however, if both *women* and *men* are (or potentially could be) members of every "*i-type*" group, this does not imply that for every "*i-type*" group $(\lambda_i > 0)$, although, this must be true for those "*i-type*" groups with the smallest "critical exposure *intensity*" levels of any. Moreover, although it seems likely that any "gender-dependent" factors for one "*i-type*" group, would also be "gender-dependent" for another, this may not be the case and it could be that some "*i-type*" *women* have a lower "critical exposure *intensity*" level compared to *men* of the same or a different "*i-type*".

Rationalization of Exposure "Intensity" when $(\lambda > 0)$ & $(R > 1)$,

Nevertheless, regardless of how we view the notion of a "critical exposure *intensity*", and regardless of the details regarding any rationalization of how such a circumstance might come to be, if we accept the proposition that both: $(\lambda > 0)$ & $(R > 1)$, there are three firm conclusions that must be incorporated into any plausible explanation of the circumstances created by this paradox. First, if *men* and *women* are (or potentially could be) members of the any particular "*i-type*" group, the hazards must be proportional within that "*i-type*" group (*see Sections 6f & 6h*). Second, if both *men* and *women* are (or potentially could be) members of every "*i-type*" group, the hazards must be proportional within the population (*see Sections 6f & 6h*). And third, as demonstrated in *Sections 4a–b* (*above*), our measure of "*intensity*", $\{H(a)\}$, is exponentially related to the probability that a susceptible *man* or *woman* (considered separately) either has or will develop MS. Also, this same measure of "*intensity*" is exponentially related to the probability that a susceptible individual (considering *men* and *women* together) either has or will develop MS and that, during any *Time Period*, this probability is a linear combination of the probabilities for *men* and *women*, considered separately (*see Section 4c, above*).

Exposure "Intensity" in Susceptible Women

Any condition for which $(\lambda > 0)$ indicates that there must be some environmental conditions in which only susceptible *men* can experience a "*sufficient*" exposure. As noted *above*, this circumstance requires that,

for at least some "*i-types*", that: $(\lambda_i > 0)$. We will define the family of exposures $\{E_{iw}\}$ to be the subset of exposures, within the $\{E_i\}$ family, that are "*sufficient*" for susceptible "*i-type*" *women* such that:

$$\forall(iw = 1,2,\ldots,m_{it}):\ \{E_{iw}\} \subset \{E_i\}$$

where, for at least one (i), it must be the case that: $P(\{E_{iw}\}) < P(\{E_i\})$

In turn, as for our earlier definition of (E) – *see Methods #1B* – we define the event (E_w) to represent the union of the (m_{it}) disjoint events, which exhibit the pairing of susceptible "*i-type*" *women* with "*sufficient*" environments, where:

$$(E_w) = (\{E_{1w}\}, G_{1t}, F) \cup (\{E_{2w}\}, G_{2t}, F) \ldots \cup (\{E_{m_{it}w}\}, G_{m_{it}t}, F)$$

and: $P(E \,|\, G, E_T) > P(E_w \,|\, G, E_T)$.

Consequently, if we adjust *Equation S8* to account for the fact that some "*i-type*" *men* require a less *"intense"* exposure than *women* of the same "*i-type*", then, the ratio, (Zw/Zm), can be re-expressed as:

$$Zw/Zm = \{P(E_w \,|\, G, F, E_T)/P(E \,|\, G, M, E_T)\} * \{\boldsymbol{d/c}\} \qquad (\textit{Equation S13})$$

6h. Exposure Variability (i.e., for R_i & λ_i) among "i-type" Individuals

If both *men* and *women* are (or potentially could be) members of any specific *i-type* group, by definition, these *men* and *women* each have a non-zero probability of developing MS in response to every one of the (v_i) "*sufficient*" sets of exposures within the $\{E_i\}$ family for this group. As discussed earlier, in these circumstances, these specific *i-types*, considered separately, will necessarily exhibit proportional hazards for the two genders (*see Section 4f; above*). We previously defined the subset $(G_{w^s}) = (F, G)$ – *see Methods #1A, Main Text* – a subset, which includes each susceptible *woman* (G_{dw^s}) in (Z), where: $(d = 1,2,\ldots,mp)$. If <u>*every*</u> *i-type* group includes (or, potentially, could include) both *men* and *women*, then, at every exposure level for a *man* $\{H(a) > \lambda\}$, we can define a proportionality constant $(R_i > 0)$, such that the exposure level for any *i-type* susceptible *woman* $\{K_i(a) > 0\}$ can be expressed as:

$$\forall\{G_{dw^s} \in (F, G_{it}):\ K_i(a) = R_i * \{H(a) - \lambda\}$$

{NB: Here, we don't need to consider the i-type specific exposure for men, $H_i(a)$, because, by definition, it is always true that, if every i-type has the same threshold $(\lambda > 0)$ then, for all $\{H(a) > \lambda\}$ and for all (i), both $\{H(a) - \lambda > 0]\}$ and $\{K_i(a) > 0\}$. Therefore, in this circumstance, there will be some constant $(R_i > 0)$ that permits this statement to be true for each (i). See below for a consideration of the impact of different i-types having different thresholds.}

Also, because each (G_{dw^s}) is a member of one or another of these posited *i-type* groups, we can define an exposure level $\{K_{dw^s}(a)\}$ and a proportionality factor $\{R_{dw^s}\}$ for each susceptible *woman* such that:

$$\forall\ G_{dw^s} \in (F, G_s):\ K_{dw^s}(a) = R_{dw^s} * (H(a) - \lambda)$$

where: $\forall\ G_{dw^s} \in (F, G_{it}):\ K_{dw^s}(a) = K_i(a)\ \ and:\ R_{dw^s} = R_i$

Thus, the expected exposure level for susceptible *women* can be expressed such that:

$$K(a) = E\{K_{dw^s}(a)\} = \sum_{d=1}^{mp} \; R_{dw^s} * \{H(a) - \lambda\}/mp = R * \{H(a) - \lambda\}$$

where: $R = E(R_{dw^s})$

Consequently, if *women* and *men* can, potentially, be members of every *i-type* group, the hazards for *women* and *men* will always be proportional, although the hazard proportionality factor (R_i) need not be the same for every *i-type* group. Additionally, it is possible that the difference in threshold between *women* and *men* (λ_i) may be different for individuals of different *i-types*. We consider, first, this possibility for those circumstance in which: $(\lambda > 0)$. In this circumstance $\{\lambda = \min(\lambda_i) > 0\}$ – at least among those "*i-types*" having the smallest "threshold" of any (*see Section 6g*) – because, by definition, some *women* will begin to develop MS at this level of exposure. We can also define the difference in threshold (λ_{dw^s}) between each susceptible *woman* and that of susceptible *men* having the smallest "threshold" (λ_m) of any. In this case, because $(\lambda > 0)$ and, by definition: $(\lambda_m = 0)$ – *see Methods #4A* – therefore:

$$\forall \; G_{dw^s} \in (F, G_{it}): \;\; \lambda_{dw^s} = \lambda_i$$

In this manner, the proportionality constants for each *i-type* $(R_i > 0)$ and each *woman* $(R_{dw^s} > 0)$ can be replaced by a *"adjusted"* proportionality constants $(R'_i > 0)$ and $(R'_{dw^s} > 0)$ such that:

$$\forall \; G_{dw^s} \in (F, G_s): \;\; K_{dw^s}(a) = R_{dw^s} * (H(a) - \lambda_{dw^s}) = R'_{dw^s} * \{H(a) - \lambda\}$$

where: $\forall \; G_{dw^s} \in (F, G_{it})$: $K_{dw^s}(a) = K_i(a)$; $R_{dw^s} = R_i$; $R'_{dw^s} = R'_i$; and: $\lambda_{dw^s} = \lambda_i$

Thus, in this case, the expected exposure level for susceptible *woman* can be expressed such that:

$$K(a) = E\{K_{dw^s}(a)\} = \sum_{d=1}^{mp} \; R'_{dw^s} * \{H(a) - \lambda\}/mp = R * \{H(a) - \lambda\}$$

where: $R'_{dw^s} = R_{dw^s} * \{(H(a) - \lambda_{dw^s})/(H(a) - \lambda)\} \leq R_{dw^s}$

and where now: $R = E(R'_{dw^s})$

For the circumstance where: $(\lambda < 0)$, the analysis is only changed in that: $\{\lambda = \max(\lambda_i)\}$ and that, in this circumstance: $(\lambda_w = 0)$. Thus, in either case, the hazards will still be proportional. By contrast, if *men* and *women* are each responding to different environmental events, the hazards will not be proportional and the response curves for *women* and *men* would need to be plotted on separate graphs, each with a different *x-axis* scale (*see Section 6g; above*). In such a circumstance, *men* with MS would be envisioned as having a disease distinct from MS in *women*. Alternatively, perhaps, it could be that, for some autosomal genotypes – e.g., (F, G_{ia}) and (M, G_{ia}) – only *women* or only *men* could be in an "*i-type*" group (G_{it}) – i.e., if either: $\{P(F, G_{ia}, G_{it}) = 0\}$ or: $\{P(M, G_{ia}, G_{it}) = 0\}$ – whereas, for other autosomal genotypes, both *men* and *women* could be members of the same "*i-type*" group (G_{it}). In this case, as noted previously, MS would then be envisioned as comprising three distinct diseases – one in *men* only, one in *women* only, and a third in both.

7. *Summary Equations for the Longitudinal Model*

7a. *Derivations*

We define three related ratios (*see Table 2; Main Text*):

$$C = P(MS)_1/P(MS)_2 \quad \text{or:} \quad P(MS)_1 = C * P(MS)_2$$

$$C_F = P(F,MS)_1/P(F,MS)_2 = C * \{P(F \mid MS)_1/P(F \mid MS)_2\}$$

$$C_M = P(M,MS)_1/P(M,MS)_2 = C * \{P(M \mid MS)_1/P(M \mid MS)_2\}$$

Using these definitions, we can derive the following *Summary Equations*.

1. First, we can re-express (Zw_2) & (Zw_1) such that:

$$Zw_2 = P(MS,E \mid G,F)_2 = P(MS \mid G,F)_2 = P(F \mid MS)_2 * \{\frac{P(MS)_2}{P(G,F)}\}$$

$$Zw_1 = P(MS \mid G,F)_1 = \frac{P(MS)_1 * P(F \mid MS)_1}{P(G,F)} = C * P(F \mid MS)_1 * \{\frac{P(MS)_2}{P(G,F)}\}$$

Therefore: $\quad Zw_2/P(F \mid MS)_2 = Zw_1/\{C * P(F \mid MS)_1\}$

so that: $\quad Zw_1 = Zw_2 * C * \{\frac{P(F \mid MS)_1}{P(F \mid MS)_2}\} = Zw_2 * \{\frac{P(MS,F)_1}{P(MS,F)_2}\} = Zw_2 * C_F \qquad$ *Equation S14a*

and similarly: $\quad Zm_1 = Zm_2 * C * \{\frac{P(M \mid MS)_1}{P(M \mid MS)_2}\} = Zm_2 * \{\frac{P(MS,M)_1}{P(MS,M)_2}\} = Zm_2 * C_M \qquad$ *Equation S14b*

Re-arrangement of *Equation S6d* (*Section 4b*) for *men* (*see above*) yields:

$$\boldsymbol{c} = \{e^{q_m} * Zm_2 - Zm_1\}/(e^{q_m} - 1)$$

and substituting for (Zm_1) from *Equation S14b* yields:

$\boldsymbol{c} = Zm_2(e^{q_m} - C_M)/(e^{q_m} - 1) \qquad$ *Equation S14c*

and similarly: $\quad \boldsymbol{d} = Zw_2(e^{q_w} - C_F)/(e^{q_w} - 1) \qquad$ *Equation S14d*

2. Also, we note that both: $(Zm_2 < \boldsymbol{c})$; and: $(Zw_2 < \boldsymbol{d})$. Therefore, from *Equation S14c* and from the definition of the ratio (C_M) – *see above* – it must be the case that:

$$Zm_2 < Zm_2 * \{e^{q_m} - C * \{P(M \mid MS)_1/P(M \mid MS)_2\}/(e^{q_m} - 1)$$

Dividing both sides of this inequality by (Zm_2) and, with rearrangement, yields:

$C < P(M \mid MS)_2/P(M \mid MS)_1 \qquad$ *Equation S14e*

and similarly: $\quad C < P(F \mid MS)_2/P(F \mid MS)_1 \qquad$ *Equation S14f*

Using the point estimates [6] that: $P(M|MS)_2 = 0.238$ & $P(M|MS)_1 = 0.315$. And inserting these values into *Equation S14e*, yields:

$$C < P(M|MS)_2/P(M|MS)_1 = 0.238/0.315 = 0.756$$

which translates to a greater than 32% increase in the penetrance of MS (or, equivalently, to a 32% increase in the prevalence of MS) between *Time Period #1* and *Time Period #2*.

3. And, finally, because: $P(MS|E,G,M) = \boldsymbol{c}$ and: $P(MS|E,G,F) = \boldsymbol{d}$; during any *Time Period*, then:

$$Zm_2 = P(MS,E|G,M)_2 = P(E|G,M)_2 * P(MS|E,G,M)$$

or: $$Zm_2 = P(E|G,M)_2 * (\boldsymbol{c})$$

with rearrangement, this becomes:

$$P(E|G,M)_2 = P(MS,E|G,M)_2/\boldsymbol{c} \qquad \textit{Equation S14h}$$

and, similarly: $$P(E|G,F)_2 = P(MS,E|G,F)_2/\boldsymbol{d} \qquad \textit{Equation S14i}$$

7b. Limits on the Value of the Parameters: $\boldsymbol{P(MS|E)}$, $(\boldsymbol{c})$ *and* $(\boldsymbol{d})$

As noted earlier (*see Sections 1a–c*), the *MZ*-twin concordance rates {i.e., $P(MS|MZ_{MS},E_T)$} may need to be converted into adjusted rates {i.e., $P(MS|IG_{MS},E_T)$} because the observed *MZ*-twin concordance rate will reflect, in part, any increased likelihood that an *MZ*-twin proband will develop MS due to the proband disproportionately sharing the (E_{twn}) and (E_{sib}) environments with a co-twin who has (or will develop) MS. However, from *Methods #1B* (*Main Text*), because *MZ*-twins don't share their (E_{pop}) environments, therefore:

whenever: $P(E)_2 \neq 1$; then also: $P(E|MZ_{MS})_2 \neq 1$

Moreover, if a susceptible proband is known to have experienced (E), then both the experience of their co-twin, and the *Time Period*, becomes irrelevant so that:

$$P(MS|E,MZ_{MS})_2 = P(MS|E)_2 = P(MS|E)$$

Therefore, assuming that, *currently*, exposure is not at its maximum – i.e., $\{P(E)_2 \neq 1\}$, then:

$$P(MS|MZ_{MS})_2 = P(MS,E|MZ_{MS})_2 = P(MS|E,MZ_{MS})_2 * P(E|MZ_{MS})_2$$

or: $$P(MS|MZ_{MS})_2 = P(MS|E) * P(E|MZ_{MS})_2$$

so that: $$P(MS|E) > P(MS|MZ_{MS})_2 \qquad \textit{Equation S15a}$$

Thus, the value of the parameter $\{P(MS|E)\}$ must be greater than the *currently* observed *MZ*-twin concordance. And similarly:

$$\boldsymbol{c} = P(MS|E,M) > P(MS|M,MZ_{MS})_2 \qquad \textit{Equation S15b}$$

and: $$\boldsymbol{d} = P(MS|E,F) > P(MS|F,MZ_{MS})_2 \qquad \textit{Equation S15c}$$

8. *Figures S1–S3*

8a. General (Common) Considerations for the Figures

Hypothetical relationships between the "critical exposure *intensity*" and disease expression that might explain the circumstance of: $(\lambda > 0)$, in which *men* disproportionately (or exclusively) experience a "*sufficient*" exposure at low "*intensity*" exposures compared to *women* (*see Section 6g; above*). In these *Figures*, the *x-axis* represents the level (or "*intensity*") of exposure and on the *y-axis* is the proportion of the susceptible population (G) who experience a "*sufficient*" exposure.

{NB: In these Figures we have plotted the theoretical exposure "intensity as a "logarithm transformed" exposure – i.e., $log(a)$ – rather than in units of cumulative hazard – i.e., $(H(a)$ – as was done for Figures 1– 4 (Main Text). Although the mapping between the (a) scale and the $H(a)$ is one-one and onto, the relationship between the two scales is non-linear – see Methods #4A (Main Text); see also Sections 4a–c, above. Moreover, the reason for using this transformation is that (a) is an odds and, therefore, the use of $log(a)$ will usually normalize the variance of these distributions [39].}

In *Panel* (A) of each *Figure*, the solid black lines represent the distribution of "*actual*" exposure "*intensity*" levels experienced by the susceptible population during a *Time Period* (E_T). The dotted lines (red for *women* and blue for *men*) represent the distributions of the "critical exposure *intensity*" (or "threshold") levels for susceptible *men* and *women* (of any "*i-type*"). These "threshold" levels are defined such that the exposure becomes "*sufficient*" for an "*i-type*" individual only once they experience an exposure "*intensity*" level, at (or above), their particular "threshold" (*see Section 6g; above*). The threshold distributions are plotted, arbitrarily, for conditions where: $(p = 0.5)$, or equivalently, under conditions where: $\{p/(1-p) = 1\}$. The blue shading in *Panel* (A) of each *Figure* represents those individuals who "*actually"* experience a "*sufficient*" exposure, at the levels of population exposure depicted. In these *Figures*, although the exposure "*intensity*" is the same for everyone, different "*i-types*" experience a "*sufficient*" exposure at different "*intensities*".

Moreover, if susceptible *men* and *women* can be plotted on the same "*intensity*" scale, this implies that both groups are responding to the same "*environmental conditions*" and that the hazards are proportional (*see Section 6g* (*above*)*; see also Methods #4A; Main Text*). Also, in this circumstance, it seems likely that the value of the proportionality constant (R) is related to a difference in these distributions of "critical exposure *intensity*" values between susceptible *men* and *women*. Therefore, it is of note that both a value of $(R > 1)$, and an increased variance in susceptible *men*, reflect the same underlying circumstance (*see Section 6g; above)* – i.e., where susceptible *men*, have a larger difference in exposure "*intensity*" between the onset of their response curve and any point on the *y-axis*, compared to *women* (e.g., *Figures 3 & 4; Main Text*).

Therefore, if this construct is correct, these two parameters – i.e., the proportionality factor (R) and the variance, as measured on the $log(a)$ scale – must be related to each other. Nevertheless, because (a) has a

non-linear relationship to $H(a)$, any exact relationship of (R) to the variance cannot be predicted. Nevertheless, if *men* and *women* have the same variance, then: $(R = 1)$ – *see Section 6g; above; see also Figure S1; Panel A & Figure 5; Main Text*.

In the *B–D Panels* of each *Figure*, we plot the cumulative probability of experiencing a "*sufficient*" environmental exposure with increasing exposure "*intensity*" (under the different conditions represented by each *Figure*). This relationship is plotted separately for susceptible *men* (solid blue lines) and *women* (solid red lines). The black lines represent the changes in the *F:M sex ratio* with increasing exposure and the scale for these lines is indicated in each *Figure*. As in the (A) *Panels* (*above*), these cumulative probability curves are mostly plotted for conditions where: $\{p/(1-p) = 1\}$. However, this choice doesn't impact the character of any response. Thus, these response curves, following a maximum exposure (e.g., *Figure 5D; Main Text*), will plateau at: $\{p/(1-p)\}$, whatever this is. Moreover, from *Equation S1a* (*Section 2c*), at any point where the *F:M sex ratio* is less than $\{p/(1-p)\}$, then, at this point, regardless of the value of (p):

$$Zw/Zm = P(MS, E \,|\, F, G)/P(MS, E \,|\, M, G) < 1$$

Also, because currently: $\{Zw/Zm > 1\}$, our current *Time Period* must be taking place after the intersection of the response curves for susceptible *men* and *women*.

In these *Figures*, the exposure level of: $\{log(a) = 0\}$, has been chosen as the point where the average odds of a "critical exposure *intensity*" is equal to (1). No other units are provided because these are undefined other than as they relate to the variance of these "critical exposure *intensity*" distributions in susceptible *men* and *women* (σ_w^2 and σ_m^2), respectively.

8b. Figures S1–S3

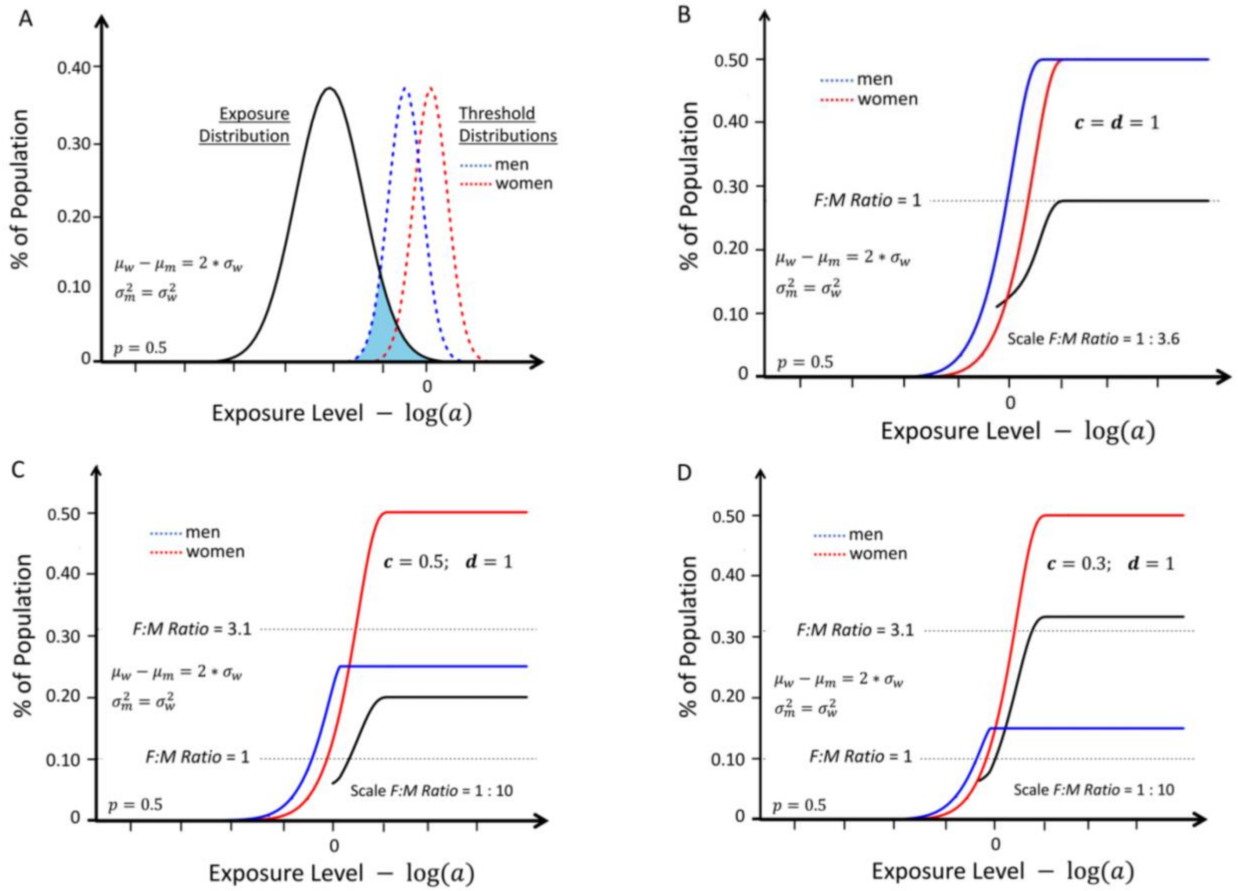

Figure S1. See the general description of the layout of *Figures S1–S3* provided in *Section 8a* (*above*). This *Figure* (*S1*) assumes that the distribution of these *log-transformed* "critical" (or threshold) levels of exposure for susceptible *men* and *women* have the same variance (σ_w^2 and σ_m^2, respectively) but different means (μ_w and μ_m, respectively) – i.e., conditions such as those depicted in *Panel A*. Because ($\lambda > 0$), and if ($\boldsymbol{c} = \boldsymbol{d} = 1$), under these conditions, a greater proportion of *men* will experience a "*sufficient*" exposure at every "*intensity*" level compared to *women* (*Panel B*). Consequently, the *F:M sex ratio* can never exceed $\{p/(1-p)\}$, and thus, in the case illustrated, this ratio cannot exceed 1. Thus, the only way to achieve the "current" proportion of *women* among MS patients (i.e., $F{:}M\ sex\ ratio = 3.1$), is for the proportion of *women* among susceptible individuals – i.e., $P(F \mid G)$ – to be greater than or equal to the current estimate for $P(F \mid MS)$ – *see Section 2c* (*above*). Such a circumstance, however, requires that $(Zm > Zw)$ throughout the entire response curve. By contrast, if conditions were such that ($\boldsymbol{c} < \boldsymbol{d} = 1$), then the response curves intersect at exposures appropriate for *Figure 4* (*Main Text*), the *F:M sex ratio* is steadily increasing throughout the response, this ratio can easily exceed its currently observed value (3.1), and there is no need to invoke any extreme conditions (e.g., *Panels C & D*). In the circumstance of these graphs, the variance is the same, which indicates that: $(R = 1)$ – *see Section 6g*. However, *Panels C & D* would be little changed if: $(R \leq 1)$ – e.g., *Panel C* of *Figure 4; Main Text*. Nevertheless, in this circumstance, the potential paradox, created by the simultaneous conditions that: $(\lambda > 0)$ and $(R > 1)$, would be avoided (*see Section 6g*).

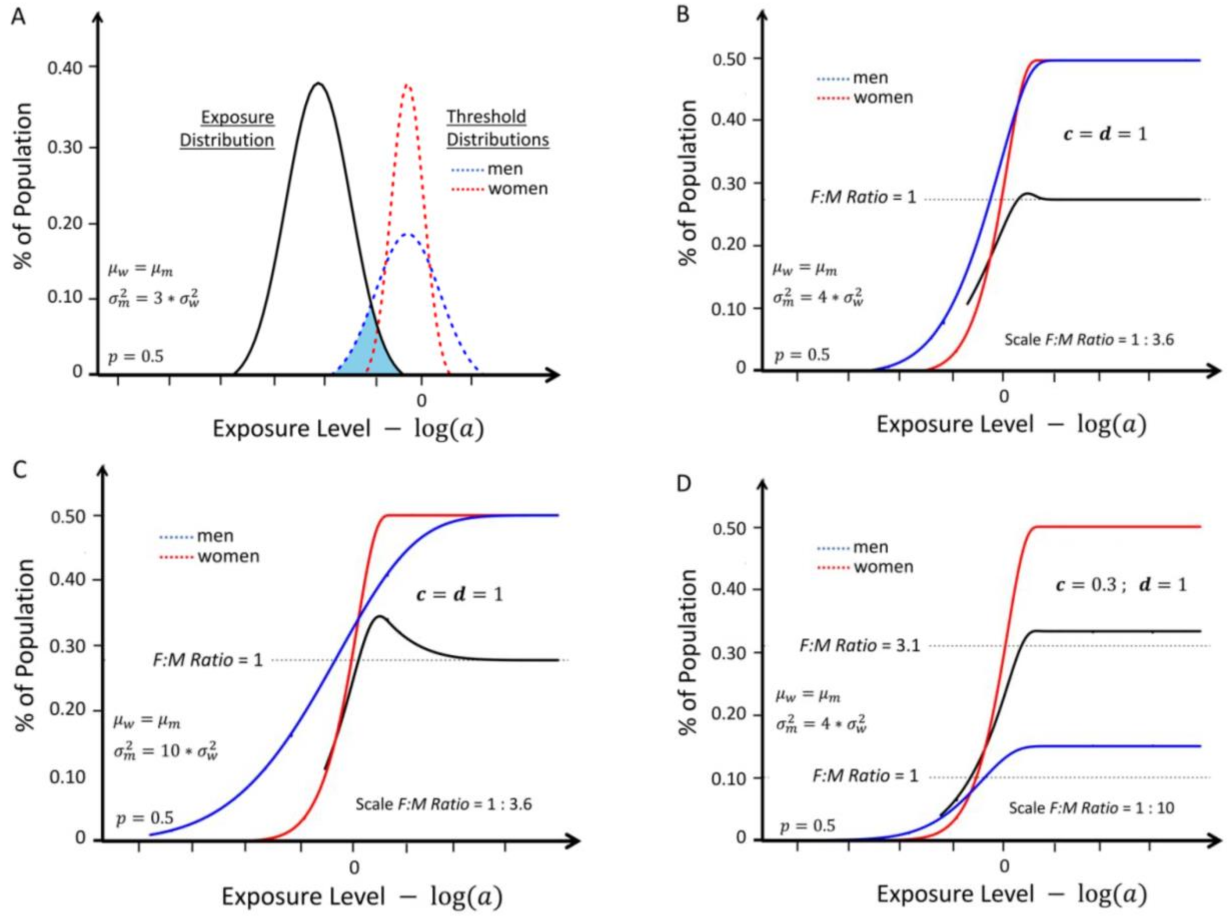

Figure S2. See the general description of the layout of *Figures S1–S3* provided in *Section 8a* (*above*). This *Figure* (*S2*) assumes that the distribution of these *log-transformed* "critical" (or threshold) levels of exposure for susceptible *women* and *men* have different variances (σ_w^2 and σ_m^2, respectively), but the same means (μ_w and μ_m, respectively) – i.e., conditions such as those depicted in *Panel A*. Under conditions where: ($\boldsymbol{c} = \boldsymbol{d} = 1$), a "*sufficient*" exposure will be experienced by a larger proportion of *men* than *women* until the response curves intersect at the point where the *F:M sex ratio* equals $\{p/(1-p)\}$. In addition, those conditions (e.g., *Panels B–C*), in which ($\boldsymbol{c} = \boldsymbol{d} = 1$), suggest that the transition from a *male-predominant* MS to *female predominant* MS takes place relatively late in the response curve for *men* – i.e., not as early or as rapidly as might be anticipated based upon the conditions presented in *Figure 3* (*Main Text*). Moreover, even though the *F:M sex ratio* can exceed the value of $\{p/(1-p)\}$, this circumstance takes rather extreme conditions (*Panels B– C*) and, for the currently observed *sex ratio* of (3.1) to be achieved [6], this would, again, require $\{p/(1-p)\}$ to be almost as large as this (*Panel C*) and for ($Zm > Zw$) until the point of intersection. Also, unlike *Figure 3* (*Main Text*), for only a small portion of these response curves is the *F:M sex ratio* declining. By contrast, if conditions were such that ($\boldsymbol{c} < \boldsymbol{d} = 1$), then, as in *Figure S1*, the response curves intersect at exposures appropriate for *Figure 4* (*Main Text*), the *F:M sex ratio* is steadily increasing throughout the response, this ratio can easily exceed its currently observed value (3.1), and there is no need to invoke any extreme conditions (e.g., *Panel D*). In this circumstance, because the variance in *men* is greater than in *women* – i.e., ($\sigma_w^2 < \sigma_m^2$) – this *Figure* only pertains to conditions of: ($R > 1$) and, thus, the paradox created by the conditions of ($\lambda > 0$) and ($R > 1$) would persist (*see Section 6g*).

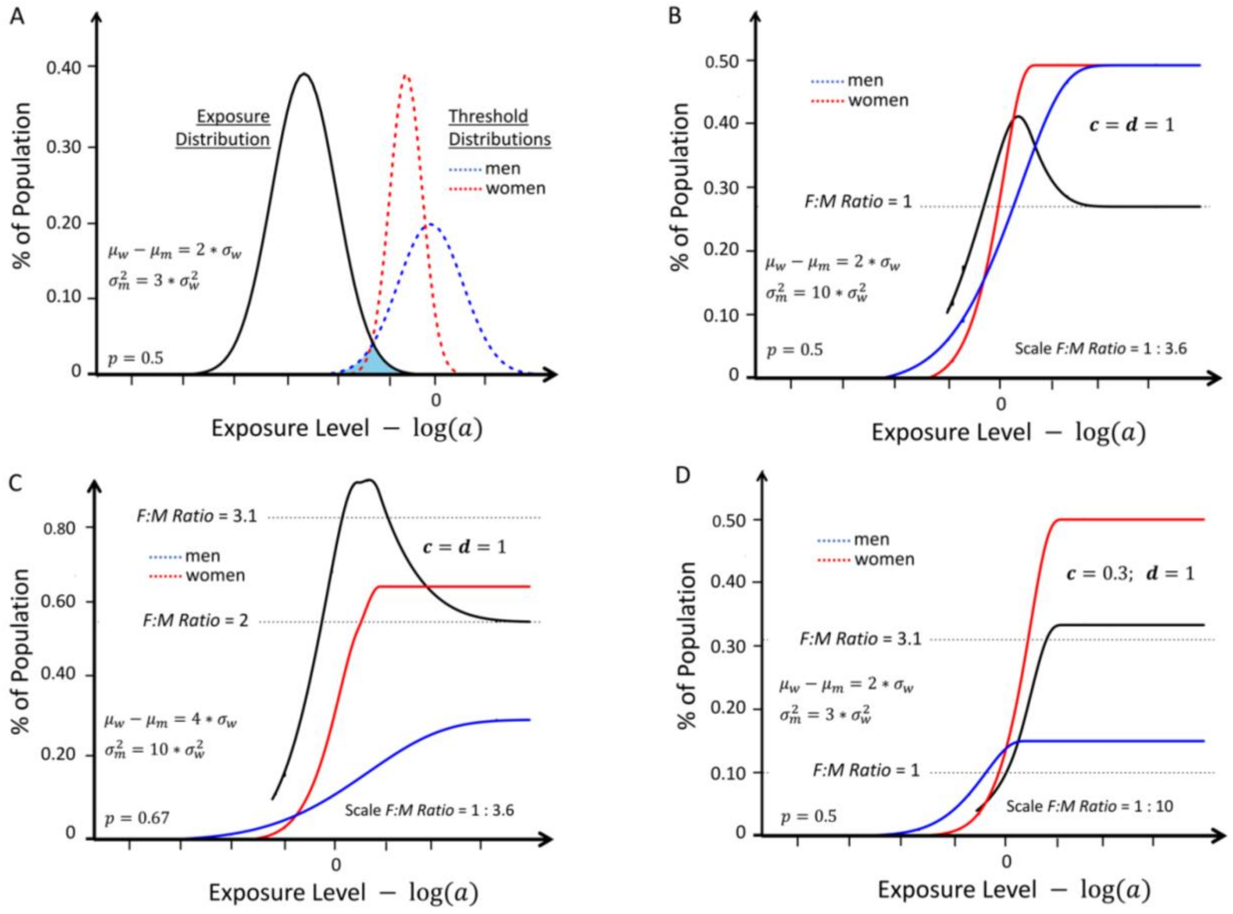

Figure S3. See the general description of the layout of *Figures S1–S3* provided in *Section 8a* (*above*). This *Figure* (*S3*) assumes that the distribution of these *log-transformed* "critical" (or threshold) levels of exposure for susceptible *women* and *men* have different variances (σ_w^2 and σ_m^2, respectively), and different means (μ_w and μ_m, respectively) – i.e., conditions such as those depicted in *Panel A*. Under conditions where: ($\boldsymbol{c} = \boldsymbol{d} = 1$), a "*sufficient*" exposure will be experienced by a larger proportion of *men* than *women* until the response curves intersect at the point where the *F:M sex ratio* equals $\{p/(1-p)\}$. In addition, those conditions (e.g., *Panels B–C*), in which ($\boldsymbol{c} = \boldsymbol{d} = 1$), suggest that the transition from a *male-predominant* MS to *female predominant* MS takes place relatively late in the response curve for *men* – i.e., not as early or as rapidly as might be anticipated based upon the conditions presented in *Figure 3* (*Main Text*). Moreover, only under extreme conditions (e.g., *Panel C*) do the response curves even approach (or surpass) the *F:M sex ratio* of (3.1), which occurs at (0.86) on the *y-axis*. Going in the opposite direction, as the difference in variance narrows, the *F:M sex ratio* does not intersect the line at the (3.1) mark until ($\sigma_m^2 = 1.3 * \sigma_w^2$). Although, in this case, the transition from a *male-predominant* MS to *female predominant* MS takes place very early the response curve, the proportion of the susceptible population who experience a "*sufficient*" exposure at the point where the curves intersect is miniscule ($i.e., < 10^{-6}$). Also, as in *Figure 3* (*Main Text*), for much of these response curves the *F:M sex ratio* is declining. By contrast, as in *Figure S2*, if conditions were such that ($\boldsymbol{c} < \boldsymbol{d} = 1$), then, as in *Figure S1*, the response curves intersect at exposures appropriate for *Figure 4* (*Main Text*), the *F:M sex ratio* is steadily increasing throughout the response, this ratio can easily exceed its currently observed value (3.1), and there is no need to invoke any extreme conditions (*Panel D*). In this circumstance, because the variance in *men* is greater than in *women* – i.e., ($\sigma_w^2 < \sigma_m^2$) – this *Figure* only pertains to conditions of: ($R > 1$) and, thus, the paradox created by the conditions of ($\lambda > 0$) and ($R > 1$) would persist (*see Section 6g*).
