# Supplementary material for: Multiple sclerosis: Exploring the limits and implications of genetic and environmental susceptibility
Source: PLoS One. 2023 Jun 28;18(6):e0285599. doi: 10.1371/journal.pone.0285599 (PMC10306391; doi:10.1371/journal.pone.0285599)
Supplement: Attachment — Submitted filename: Response to Reviewers.doc [file pone.0285599.s002.doc]

# UNIVERSITY OF CALIFORNIA, SAN FRANCISCO

| BERKELEY · DAVIS · IRVINE · LOS ANGELES · MERCED · RIVERSIDE · SAN DIEGO · SANTA CRUZ |  | SANTA BARBARA · SAN FRANCISCO |
| --- | --- | --- |

| Douglas S. Goodin, MD  Professor of Neurology | Department of Neurology, Rm. M794  San Francisco, CA 94143-0114  Tel: (415) 514-2464 Fax: (415) 514-2470  E-mail: douglas.goodin@ucsf.edu |
| --- | --- |
|  |  |

5 February 2023

Luwen Zhang, *Academic Editor*
PLOS ONE

Dear Dr. Zhang:

Re:	Multiple Sclerosis: Exploring the Limits and Implications of Genetic and Environmental Susceptibility

Goodin DS, Khankhanian P, Gourraud PA, Vince N

Thank you very much for your e-mail of 2 January 2023 and for your invitation to submit a revision of the above referenced manuscript. We have been over the manuscript with the comments of the Reviewer in mind and have amended the manuscript accordingly. We detail (below) the modifications that we have made.

Reviewer #1

*Comments*

*1. The format is not attuned to the PlosOne format and readers. About 90% of the sections “Methods” and “Longitudinal model” should be moved to supplementary material. The Discussion section should be split: the math-heavy part should go into supplementary, and the more clinical part should be retained in the main text. The Conclusion section is missing – even if it is a half-page, I’d ask authors to please add it.*

*Response:*

As requested, we have removed a large portion of the mathematical development from the *Methods* and *Discussion* sections of the *Main Text* and placed this material into a new *Supplementary Material*. We have also added a *Conclusion* section, as requested.

*2. While the statistical part is extremely well detailed, other prior work on stochasticity in MS that came up in the past 10 years, have not been cited. At minimum, authors should incorporate these 8 references from this query.*

*Response:*

As requested, we have incorporated a discussion of these additional papers into the *Discussion* section of the *Main Text*.

I hope with these modifications, you will now find the manuscript suitable for publication in *PLoS One*. Thank you very much for your help and your consideration of this matter. I look forward to hearing from you in due course.

Yours sincerely,

Douglas S. Goodin, MD

*Professor of Neurology*

*University of California, San Francisco*
